# Supplementary material for: CRISPR-Cas9-based non-viral gene editing therapy for topical treatment of recessive dystrophic epidermolysis bullosa
Source: Mol Ther Methods Clin Dev. 2023 Oct 13;31:101134. doi: 10.1016/j.omtm.2023.101134 (PMC10630779; doi:10.1016/j.omtm.2023.101134)
Supplement: Document S1. Figures S1–S10 and Tables S1–S3 [file mmc1.pdf]

OMTM, Volume *31*

Supplemental information

CRISPR-Cas9-based non-viral gene editing therapy for topical treatment of recessive dystrophic epidermolysis bullosa

Xianqing Wang, Xi Wang, Yinghao Li, Sigen A, Bei Qiu, Albina Bushmalyova, Zhonglei He, Wenxin Wang, and Irene Lara-Sáez

Supplemental Information

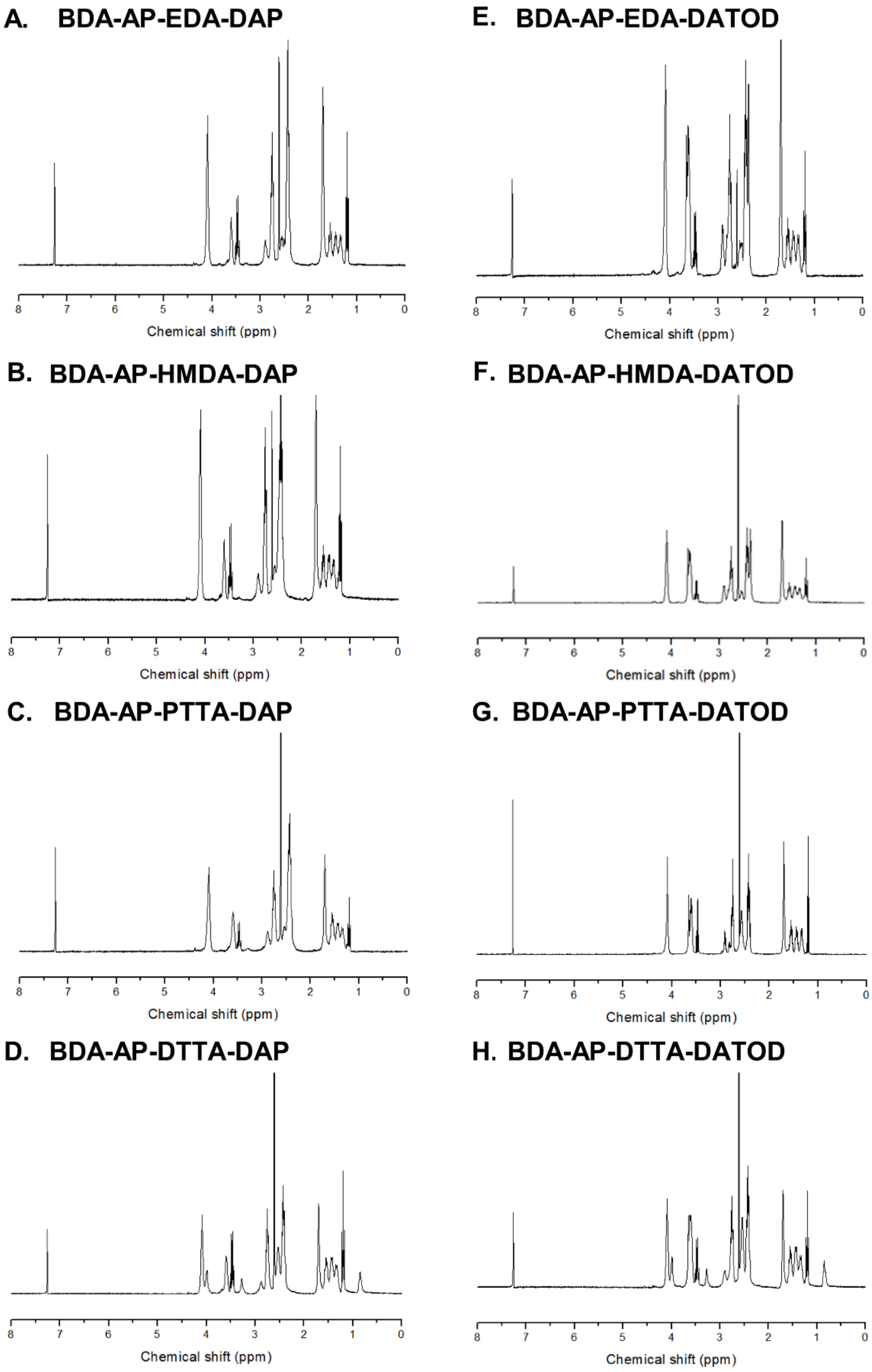

Figure S1 ^{1}H NMR spectrum of HPAEs (400 MHz, CDCl3). (A)BDA-AP-EDA-DAP; (B) BDA-AP-HMDA-DAP; (C)BDA-AP-PTTA-DAP; (D) BDA-AP-DTTA-DAP; (E) BDA-AP-EDA-DATOD; (B) BDA-AP-HMDA-DATOD; (C)BDA-AP-PTTA-DATOD; (D) BDA-AP-DTTA-DATOD.

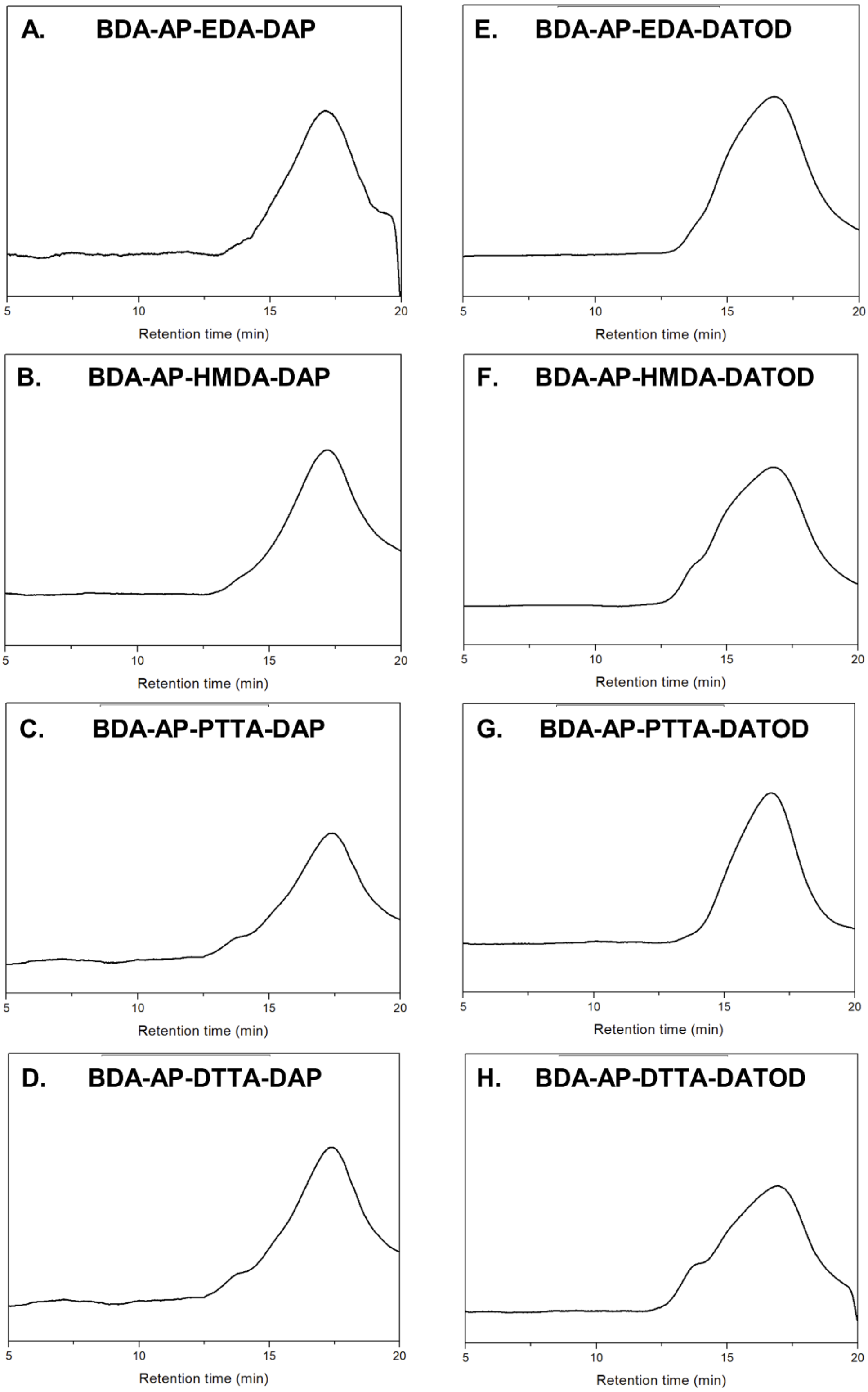

Figure S2 GPC retention time traces of HPAEs. (A)BDA-AP-EDA-DAP; (B) BDA-AP-HMDA-DAP; (C)BDA-AP-PTTA-DAP; (D) BDA-AP-DTTA-DAP; (E) BDA-AP-EDA-DATOD; (B) BDA-AP-HMDA-DATOD; (C)BDA-AP-PTTA-DATOD; (D) BDA-AP-DTTA-DATOD.

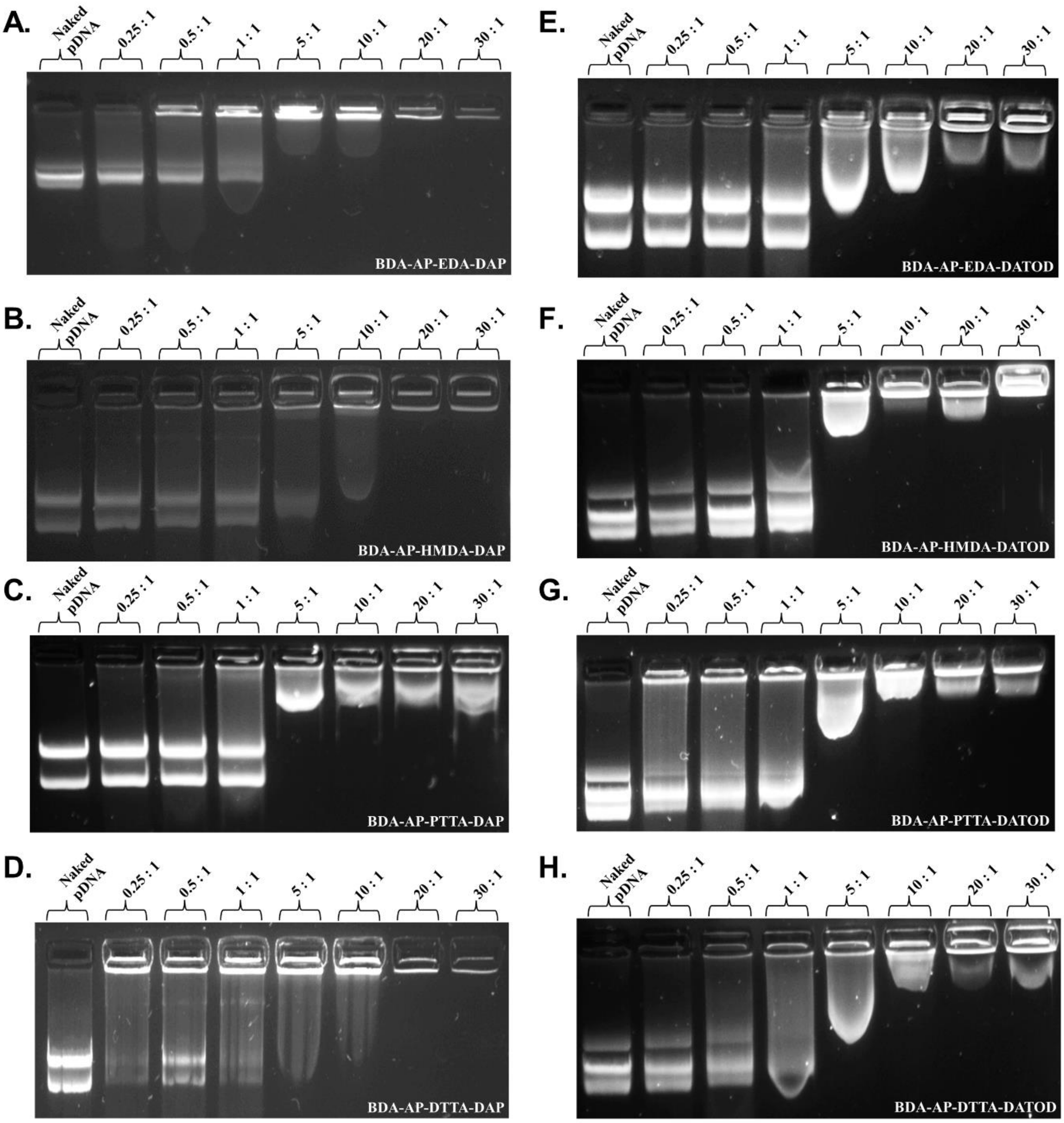

Figure S3 Gel retardation of HPAE/gWiz GFP pDNA. (A)BDA-AP-EDA-DAP; (B) BDA-AP-HMDA-DAP; (C)BDA-AP-PTTA-DAP; (D) BDA-AP-DTTA-DAP; (E) BDA-AP-EDA-DATOD; (B) BDA-AP-HMDA-DATOD; (C)BDA-AP-PTTA-DATOD; (D) BDA-AP-DTTA-DATOD. The DNA binding ability was tested at HPAE to DNA weight ratios from 0.25:1 to 30:1.

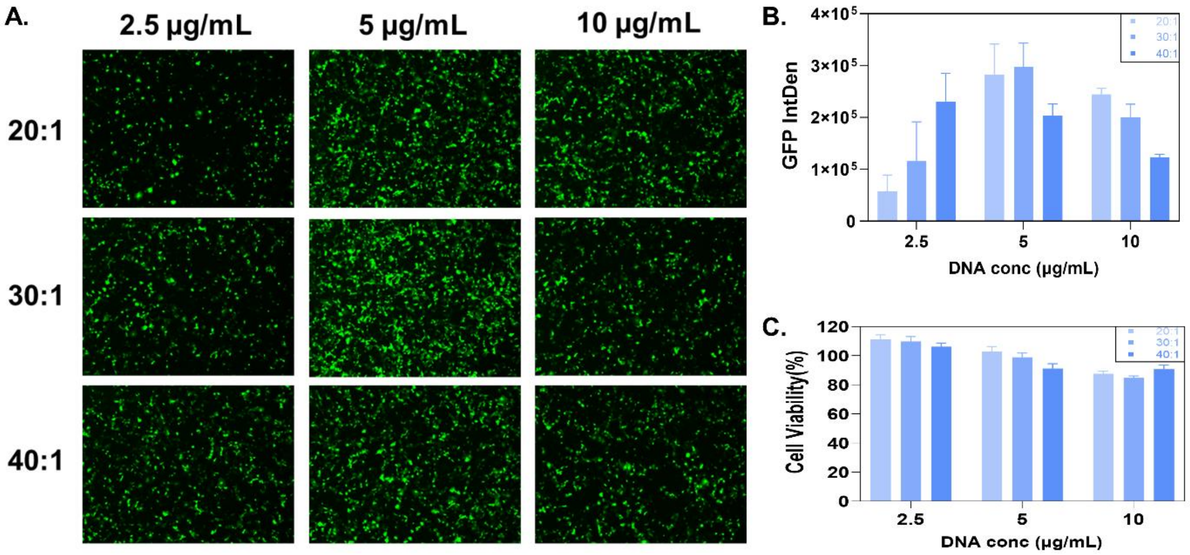

Figure S4 Transfection condition optimization of PTTA-DATOD/GFP pDNA on RDEB keratinocytes. All the data was obtained from 3 individual experiments and presented as Mean ± SD (n=3).

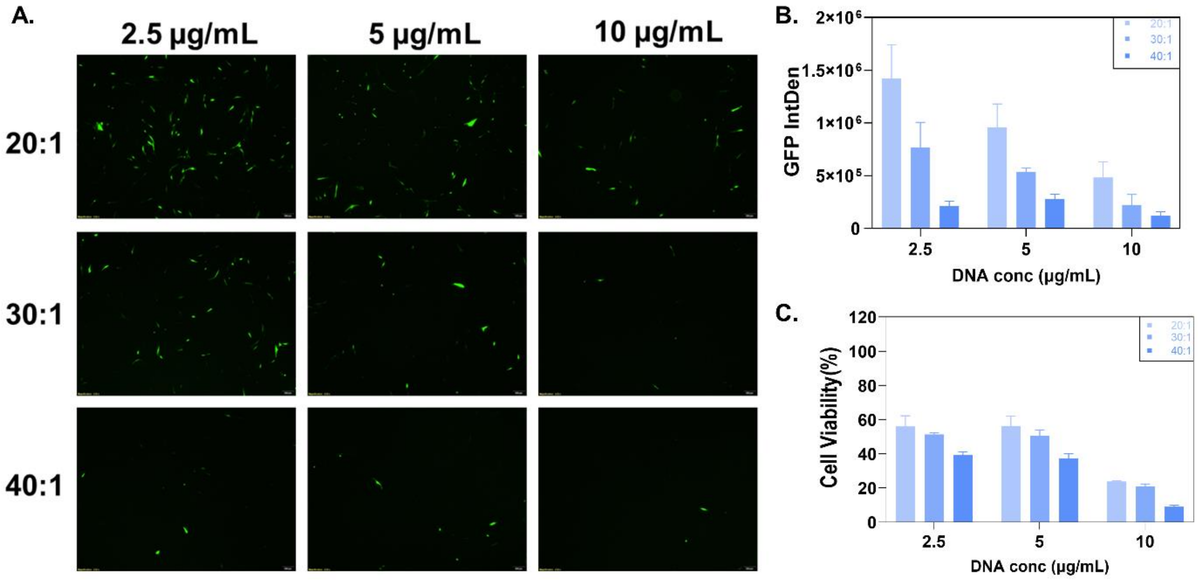

Figure S5 Transfection condition optimization of PTTA-DATOD/GFP pDNA on RDEB Fibroblasts. All the data was obtained from 3 individual experiments and presented as Mean ± SD (n=3).

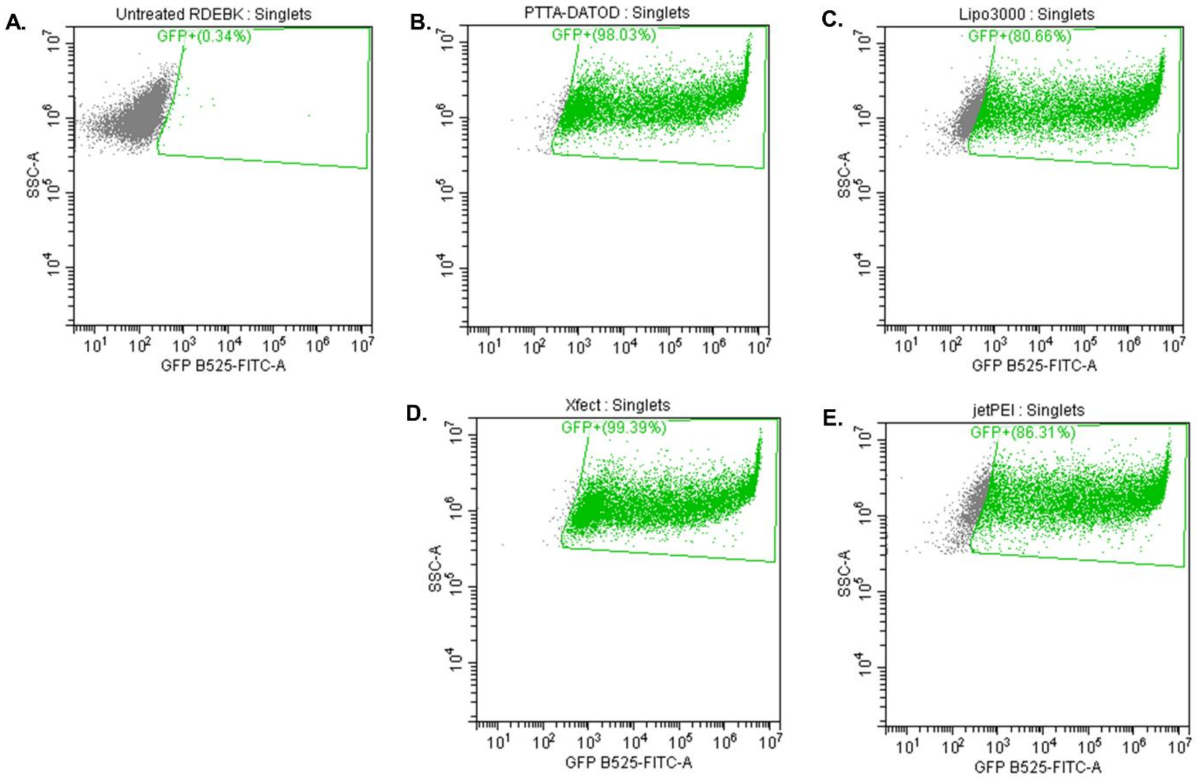

Figure S6 Gating strategy for flowcytometry analysis of RDEBK cells transfected by HPAE/gWiz GFP DNA. (A) Untreated RDEBK cells; (B) RDEBK cells treated with PTTA-DATOD/GFP DNA complexes; (C) RDEBK cells treated with Lipofectamine 3000/GFP DNA complexes; (D) RDEBK cells treated with Xfect/GFP DNA complexes; (E) RDEBK cells treated with jetPEI/GFP DNA complexes.

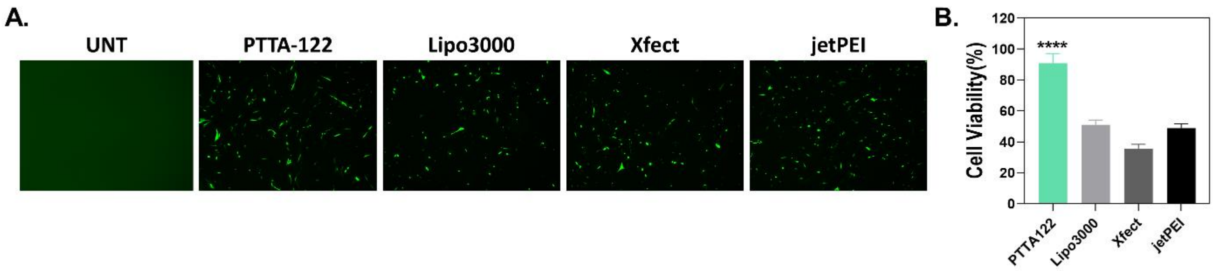

Figure S7 gWiz GFP pDNA transfection in RDEBF cells. (A) Fluorescence images of RDEBF cells transfected by PTTA-DATOD, lipofectamine 3000 (Lipo3000), Xfect and jetPEI (from left to right); (B) Cell viability of treated RDEBF cells. Data was presented as Mean ± SD (n=3).

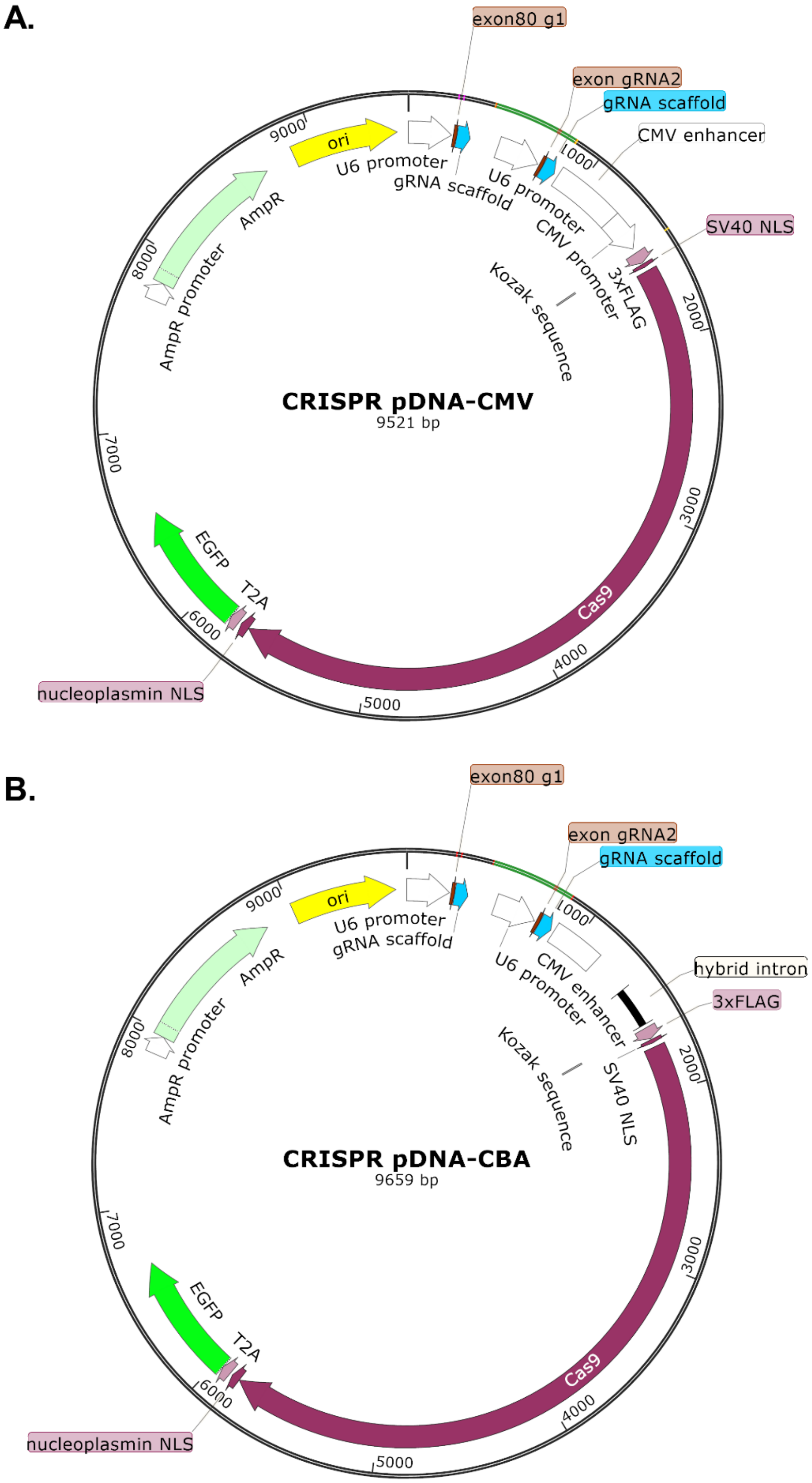

Figure S8 Maps of dual sgRNA guided CRISPR Cas9 plasmids.

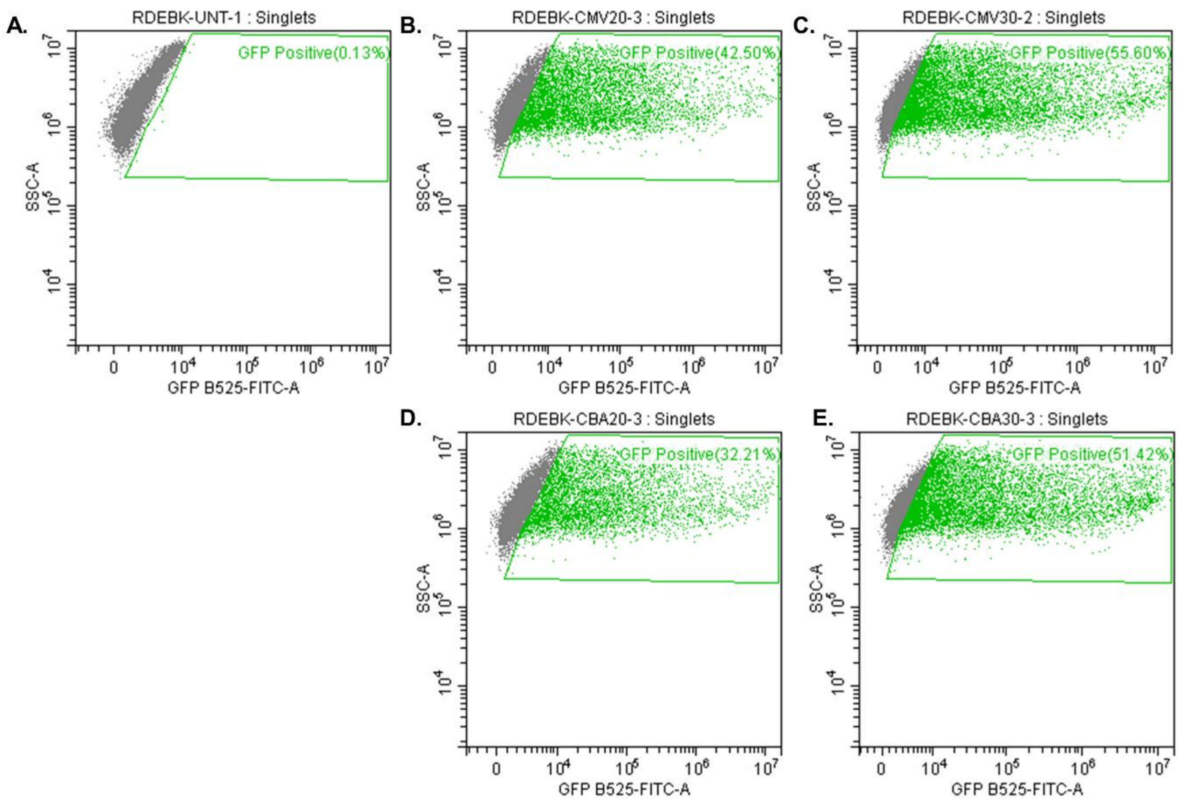

Figure S9 Gating strategy for flowcytometry analysis of RDEBK cells transfected by PTTA-DATOD/CRISPR DNA. (A) Untreated RDEBK cells; (B) RDEBK cells treated with PTTA-DATOD/CRISPR DNA at W/W of 20, the CRISPR DNA has CMV promoter; (C) RDEBK cells treated with PTTA-DATOD/CRISPR DNA at W/W of 30, the CRISPR DNA has CMV promoter; (D) RDEBK cells treated with PTTA-DATOD/CRISPR DNA at W/W of 20, the CRISPR DNA has CBA promoter; (E) RDEBK cells treated with PTTA-DATOD/CRISPR DNA at W/W of 30, the CRISPR DNA has CBA promoter.

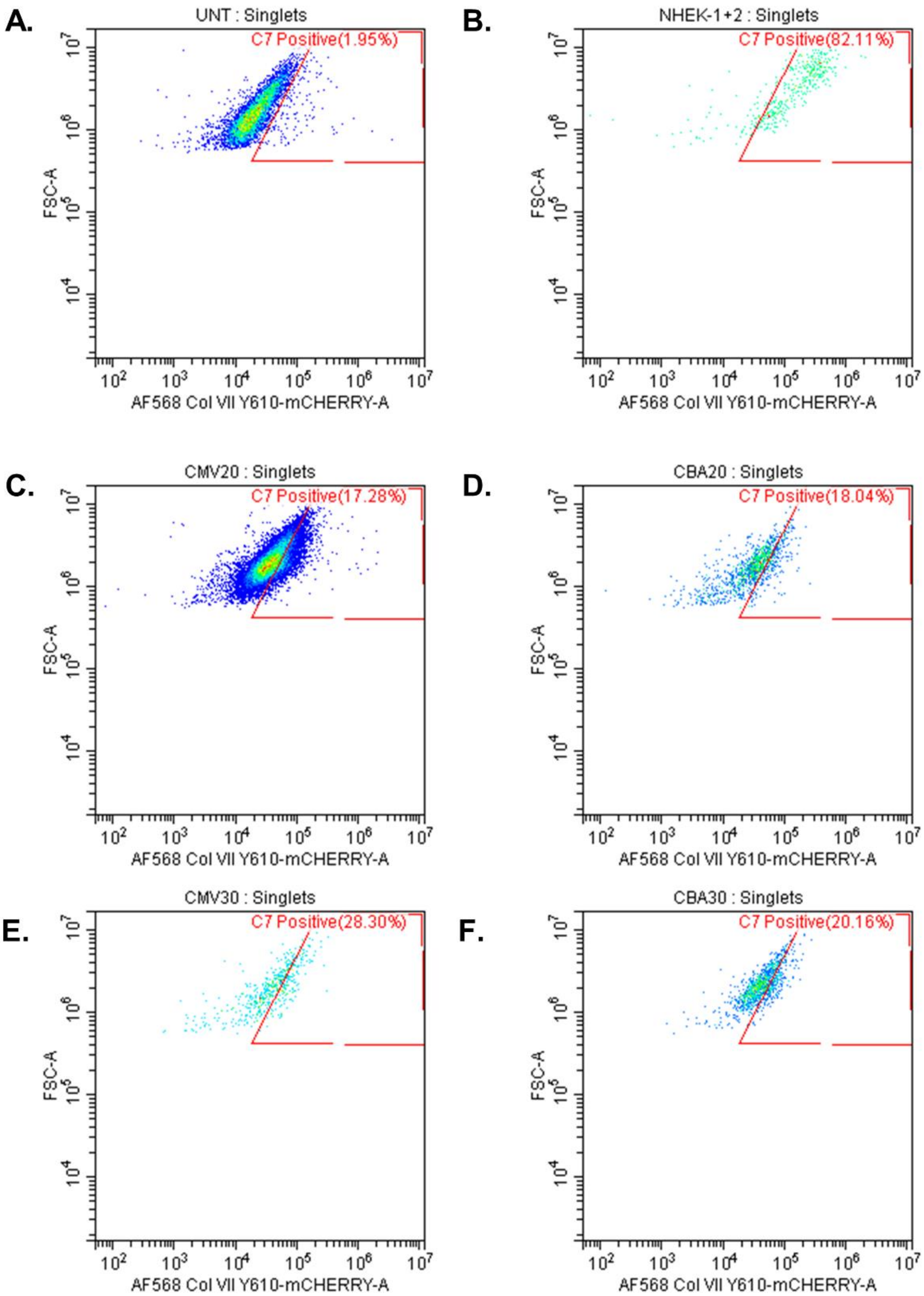

Figure S10 Gating strategy for flow cytometry analysis of type VII collagen expression in cells (A) Untreated RDEBK cells; (B) Human normal epidermal keratinocytes; (C) RDEBK cells treated with PTTA-DATOD/CRISPR DNA at W/W of 20, the CRISPR DNA has CMV promoter; (D) RDEBK cells treated with PTTA-DATOD/CRISPR DNA at W/W of 30, the CRISPR DNA has CMV promoter; (E) RDEBK cells treated with PTTA-DATOD/CRISPR DNA at W/W of 20, the CRISPR DNA has CBA promoter; (F) RDEBK cells treated with PTTA-DATOD/CRISPR DNA at W/W of 30, the CRISPR DNA has CBA promoter.

Table S1 GPC Results

Polymer	Mn	Mw	PDI
BDA-AP-EDA-DAP	2576	8542	3.32
BDA-AP-EDA-DATOD	3481	13408	3.85
BDA-AP-HMDA-DAP	2478	8256	3.33
BDA-AP-HMDA-DATOD	3528	17068	4.84
BDA-AP-PTTA-DAP	2409	10716	4.45
BDA-AP-PTTA-DATOD	3500	10007	2.86
BDA-AP-DTTA-DAP	3297	38016	11.53
BDA-AP-DTTA-DATOD	5172	46832	9.05

Table S2 CRISPR Cas9 sgRNA sequence

sgRNA name	Sequence (5'-3')
sgRNA 1	CCTGCAGACCCTACATAGAG
sgRNA 2	CAAGACAGGTGAAGGTTCTT

Table S3 TaqMan™ probes for RT-qPCR

Target gene	Probe
Human COL7A1 exon 64	Hs00164310_m1
Human COL7A1 exon 80	Hs01574801_g1
Human GAPDH	Hs02758991_g1
