# Supplementary material for: CRISPR-Cas9-based non-viral gene editing therapy for topical treatment of recessive dystrophic epidermolysis bullosa
Source: Mol Ther Methods Clin Dev. 2023 Oct 13;31:101134. doi: 10.1016/j.omtm.2023.101134 (PMC10630779; doi:10.1016/j.omtm.2023.101134)
Supplement: Document S2. Article plus supplemental information [file mmc2.pdf]

Molecular Therapy
Methods & Clinical Development
Original Article

CRISPR-Cas9-based non-viral gene editing therapy for topical treatment of recessive dystrophic epidermolysis bullosa

Xianqing Wang,[1] Xi Wang,[2] Yinghao Li,[1] Sigen A,[2] Bei Qiu,[1] Albina Bushmalyova,[1] Zhonglei He,[2] Wenxin Wang,[1,2] and Irene Lara-Sáez[1]

[1]Charles Institute of Dermatology, School of Medicine, University College Dublin, D04 V1W8 Dublin, Ireland; [2]Research and Clinical Translation Center of Gene Medicine and Tissue Engineering, School of Public Health, Anhui University of Science and Technology, Huainan 232001, China

Recessive dystrophic epidermolysis bullosa (RDEB) is an autosomal monogenic skin disease caused by mutations in *COL7A1* gene and lack of functional type VII collagen (C7). Currently, there is no cure for RDEB, and most of the gene therapies under development have been designed as *ex vivo* strategies because of the shortage of efficient and safe carriers for gene delivery. Herein, we designed, synthesized, and screened a new group of highly branched poly(β amino ester)s (HPAEs) as non-viral carriers for the delivery of plasmids encoding dual single-guide RNA (sgRNA)-guided CRISPR-Cas9 machinery to delete *COL7A1* exon 80 containing the c.6527dupC mutation. The selected HPAEs (named PTTA-DATOD) showed robust transfection efficiency, comparable with or surpassing that of leading commercial gene transfection reagents such as Lipofectamine 3000, Xfect, and jetPEI, while maintaining negligible cytotoxicity. Furthermore, CRISPR-Cas9 plasmids delivered by PTTA-DATOD achieved efficient targeted deletion and restored bulk C7 production in RDEB patient keratinocyte polyclones. The non-viral CRISPR-Cas9-based *COL7A1* exon deletion approach developed here has great potential to be used as a topical treatment for RDEB patients with mutations in *COL7A1* exon 80. Besides, this therapeutic strategy can easily be adapted for mutations in other *COL7A1* exons, other epidermolysis bullosa subtypes, and other genetic diseases.

INTRODUCTION

Epidermolysis bullosa (EB) is an inherited rare genetic skin disorder, and one of its most devastating subtypes is recessive dystrophic EB (RDEB), caused by biallelic mutations in the *COL7A1* gene. Those mutations result in loss of function, deficiency, or absence of wild-type type VII collagen (C7) and disrupted anchoring fibrils in patients' skin. Patients with RDEB suffer from skin blistering, mucocutaneous scarring, milia formation, fibrosis, and high risk for developing cutaneous squamous cell carcinomas (SCCs), leading to premature death.[1–3] Currently, RDEB patients rely mainly on multidisciplinary palliative care to minimize the risk for blister formation, infection, and SCCs. The first therapy recently approved by the U.S. Food and Drug Administration (FDA) for patients with RDEB, known as beremagene geperpavec (B-VEC),[4] is a topical gene replacement therapy using engineered herpes simplex virus 1 (HSV-1) as a gene delivery vector.

With the rapid development of gene editing tools, highly specific gene editing for patients has attracted more attention and been achievable. The gene editing therapies under development for RDEB can be briefly classified into two types. One is RNA modulation using small interfering RNA (siRNA),[5] antisense oligonucleotides (AONs),[6,7] and spliceosomes.[8,9] The other strategy is genome editing mediated by gene editing nucleases such as meganuclease,[10] transcription activator-like effector nuclease (TALEN),[11,12] and CRISPR-Cas9 machinery.[13–19] Almost all those gene editing therapies are designed as *ex vivo* applications because of a lack of safe and efficient methods for *in vivo* delivery of the gene editing materials.

For the intracellular delivery of cargos required in gene therapy, there are two main approaches[20]: physical methods based on membrane disruption internalization and carrier-mediated approaches that can be further divided into viral- and non-viral-based delivery. Physical methods, such as electroporation,[21] gene gun,[22] and micro-injection,[23] are difficult to directly apply *in vivo*, particularly for RDEB patients, who have fragile skin. Compared with viral vectors, non-viral vectors are more attractive to clinical application,[24,25] as they have some advantages versus viruses: (1) high cargo encapsulation capacity; (2) low immunogenicity, genotoxicity, and oncogenicity; and (3) simpler and more economical manufacturing.

Among non-viral gene delivery vectors, poly(β amino ester)s (PAEs) are among the promising cationic polymeric carriers because of their biodegradation in physiological environments and high performance in gene delivery. Since 2003,[26] when PAEs for DNA delivery were first

Received 7 June 2023; accepted 11 October 2023;
https://doi.org/10.1016/j.omtm.2023.101134.

Correspondence: Wenxin Wang, Charles Institute of Dermatology, School of Medicine, University College Dublin, D04 V1W8 Dublin, Ireland.
E-mail: wenxin.wang@ucd.ie
Correspondence: Irene Lara-Sáez, Charles Institute of Dermatology, School of Medicine, University College Dublin, D04 V1W8 Dublin, Ireland.
E-mail: irene.lara-saez@ucd.ie

This is an open access article under the CC BY-NC-ND license (http://creativecommons.org/licenses/by-nc-nd/4.0/).

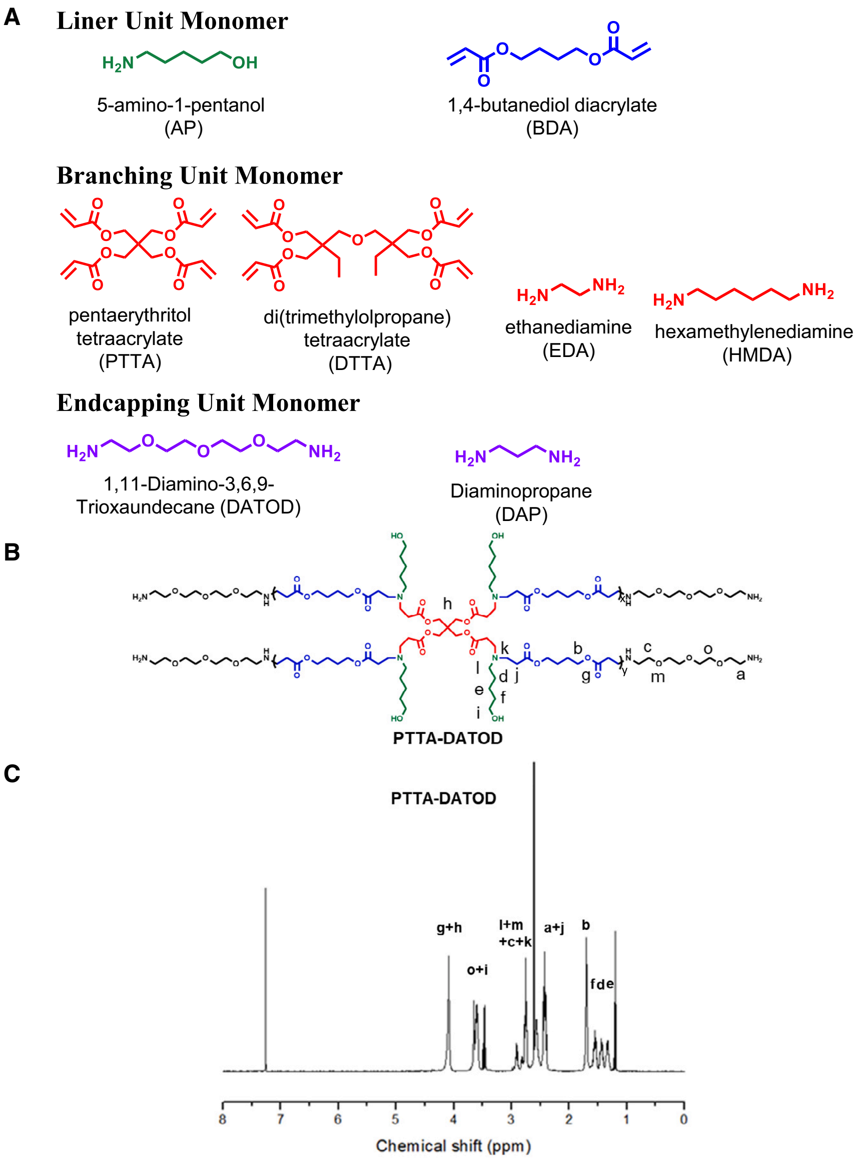

Figure 1. Highly branched poly(β-amino ester)s (HPAEs) synthesis

(A) Structure of the monomers used for the synthesis of HPAEs in this work. (B) Scheme of PTTA-DATOD structure. (C) ^{1}H-NMR spectra of PTTA-DATOD.

reported by Langer and co-workers, they have been widely used in the gene delivery for various genetic diseases.[27,28] In 2016, our group first achieved highly branched PAEs (HPAEs) synthesis via a facile "A2+B3+C2" Michael addition strategy, enhancing the performance of PAEs in gene transfection in different types of cells *in vitro*.[29] HPAEs' outstanding gene delivery ability was also proved *in vivo*, by delivering plasmids encoding full *COL7A1* gene into both *COL7A1* knockout and RDEB xenograft mouse models.[29,30]

In this work, we designed and synthesized a group of new HPAEs using a "A2+B4+C2" Michael addition strategy to deliver plasmids that encode a dual single-guide RNA (sgRNA)-guided CRISPR-Cas9 machinery for deletion of human *COL7A1* exon 80 containing the c.6527dupC mutation. The c.6527dupC mutation is prevalent in 43% of the Spanish RDEB alleles and leads to a premature codon termination resulting in a complete absence of C7.[31] Bonafont and co-workers have provided proof of principle for the deletion of *COL7A1* exon 80 containing the c.6527dupC mutation as a potential RDEB treatment,[16,32,33] on the basis of experience from exon skipping therapy for RDEB[34] and functional studies on the truncated C7.[35,36] They first developed an *ex vivo* therapy using TALEN delivered by adenoviral vectors, for whole exon 80 deletion in RDEB patient epidermal stem cells carrying the homozygous c.6527dupC mutation.[32] The *COL7A1* frame restoration efficiency was further improved via non-homologous end-joining (NHEJ) using CRISPR-Cas9 ribonucleoprotein that was electroporated into primary RDEB patient keratinocytes (RDEBK) as *ex vivo* treatment.[33] More recently, the same group used adenoviral vectors for topical delivery of CRISPR-Cas9 machinery to delete *COL7A1* exon 80 in RDEB xenograft mouse model *in vivo*.[16] Herein, to avoid the drawbacks from virus vectors, we used HPAEs as delivery carriers for the CRISPR-Cas9 system to develop a potential topical treatment for RDEB patients having mutations in *COL7A1* exon 80.

RESULTS

HPAEs synthesis and characterization

HPAEs polymers were synthesized by using 1,4-butanediol diacrylate (BDA) and 5-amino-1-pentanol (AP) as a linear unit, combined with a diamine (ethanediamine [EDA] or hexamethylenediamine [HMDA]) or tetraacrylate (pentaerythritol tetraacrylate [PTTA] or di[trimethylolpropane] tetraacrylate [DTTA]) as the branching units and further end-capped with diaminopropane (DAP) or 1,11-diamino-3,6,9-trioxaundecane (DATOD) (Figure 1). This synthesis produced 8 different branched HPAEs: EDA-DAP, HMDA-DAP, PTTA-DAP, DTTA-DAP, EDA-DATOD, HMDA-DATOD, PTTA-DATOD, and DTTA-DATOD (Table S1). From the ^{1}H-NMR (nuclear magnetic resonance) results (Figures 1 and S1), no acrylate peak can be observed in all samples after the end-capping reaction. The molecular weight (Mw) of those polymers ranged from 8 to 46 kDa. In the EDA and HMDA groups, the Mw of DATOD end-capped polymers was higher than that of DAP end-capped polymer. In the PTTA group, the Mw of both was 10 kDa. In the DTTA group,

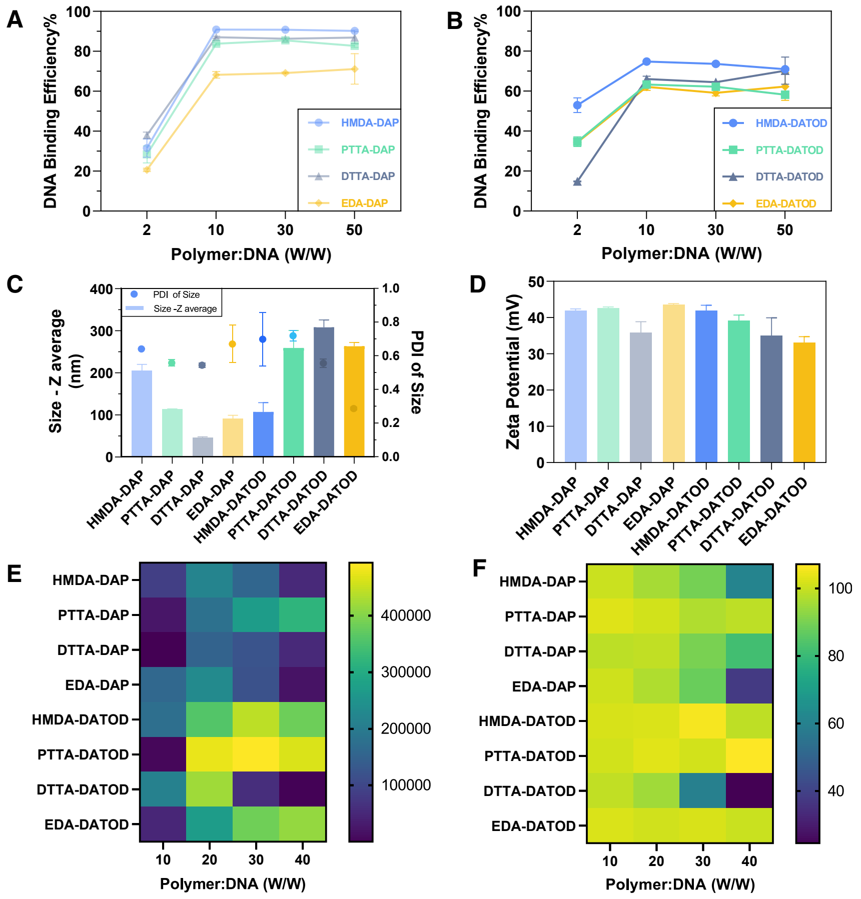

Figure 2. HPAEs/GFP pDNA complexes characterization and transfection results

(A) DNA binding efficiency of HMDA-DAP, PTTA-DAP, DTTA-DAP, and EDA-DAP at W/W ranging from 2 to 50. (B) DNA binding efficiency of HMDA-DATOD, PTTA-DATOD, DTTA-DATOD, and EDA-DATOD at W/W ranging from 2 to 50. (C) Size of HPAEs/GFP pDNA complexes at W/W of 20. (D) Zeta potential of HPAEs/GFP pDNA complexes at W/W of 20. (E) GFP fluorescence intensity from HEK cells treated by HPAEs/GFP pDNA complexes at W/W ranging from 10 to 40, at 48 h after transfection. Fluorescence intensity was semi-quantified using ImageJ Fiji. (F) Cell viability of HEK cells treated with HPAEs/GFP pDNA complexes at W/W ranging from 10 to 40, at 48 h after transfection. All data were obtained from 3 individual experiments, normalized to the untreated RDEBK, and presented as mean ± SD (n = 3).

the Mw was much higher than the other groups, ranging from 38 to 47 kDa, presumably because of the high reaction activity of DTTA monomer.

Nanoparticle formulation and gene delivery

For investigation of DNA binding capabilities, HPAEs were self-assembled with gWiz-GFP plasmid (GFP pDNA) into nanoparticles. At a ratio of HPAEs to pDNA weight (W/W) of 10, all HPAEs achieved the maximum DNA binding efficiency (BE), whereas the DAP end-capped HPAEs group had higher BE (ranging from 70% to 90%) than the DATOD group, with maximum 70% efficiency (Figures 2A and 2B). In both terminal groups, the HMDA branching monomer contributed to highest BE, while PTTA and DTTA showed similar and moderate behaviors, and EDA had lowest BE. The results from agarose gel electrophoresis retardation assay (Figure S3) also confirmed the DNA binding capabilities of those HPAEs. It is worth noting that the DAP end-capped HPAEs group not only encapsulated more DNA than the DATOD group, but the interaction between the DAP end-capped HPAEs and DNA was also stronger. For nanoparticle characterization, all the HPAEs formed nanoparticles with GFP pDNA at W/W of 20, which was the best transfection W/W in our previous study.[37] Those nanoparticles measured 45–300 nm in hydrodynamic diameter, with a polydispersity index (PDI) less than 1.0, and presented positive surface charge of about 40 mV (Figures 2C and 2D). The DAP end-capped group with stronger binding capabilities led to smaller nanoparticle size than the DATOD group, except the HMDA-DAP polymer, which had about 200 nm diameter.

GFP expression was first analyzed in human embryonic kidney 293 (HEK) cells at W/W ranging from 10 to 40. Surprisedly, DATOD end-capped group showed GFP gene expression twice as high as that of the DAP end-capped group (Figure 2E). Among the DATOD and DAP end-capped groups, PTTA-DATOD and HMDA-DATOD had outstanding highest GFP expression levels over the other six HPAEs ($p < 0.001$), while PTTA-DAP performed best in the DAP end-capped group ($p < 0.001$). From the quantitative data shown in Figure 4F, cell viability only diminished below 70% with DTTA-DATOD at W/W of 30 and HMDA-DAP, EDA-DAP, and DTTA-DATOD at W/W of 40. Considering the gene expression level and cytotoxicity of those HPAEs in mediating gene transfection, PTTA-DATOD was selected for further experiments in RDEB cells.

HPAEs in gene delivery in RDEB cells

In normal human skin, C7 is produced mainly by epidermal keratinocytes and partly by dermal fibroblasts. To further evaluate the suitability of PTTA-DATOD as a gene carrier for RDEB gene therapy, GFP pDNA was transfected into immortalized RDEBK and RDEB patient fibroblasts (RDEBF). As shown in Figures S4 and S5, PTTA-DATOD/GFP pDNA had the best performance at pDNA concentration of 5 μL mL^{-1} in RDEBK, and 2.5 μL mL^{-1} pDNA was the best concentration for RDEBF. Although no obvious effect in cell viability was observed under each condition, a tendency was observed that the cell viability decreased with increasing pDNA concentration and W/W ratios (Figures S4C and S5C).

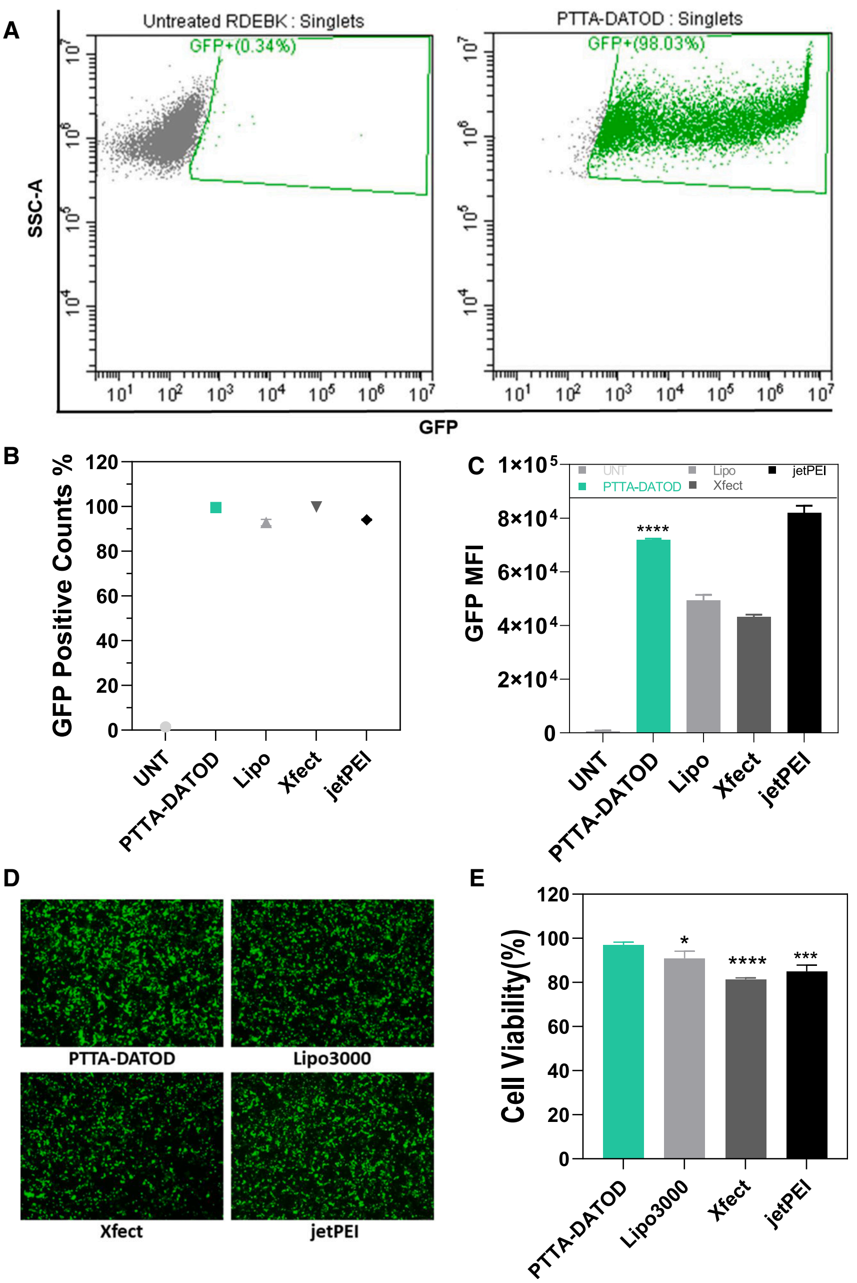

Figure 3. Transfection efficiency analysis of PTTA-DATOD/GFP pDNA transfected RDEBK

RDEBK were treated with PTTA-DATOD/GFP pDNA complexes at W/W of 30 and after 24 h transfection. (A) GFP-positive cell population, (B) GFP-positive counts, and (C) GFP mean fluorescence intensity (MFI) were quantified using flow cytometry. Lipo3000, Xfect, and jetPEI were used as controls. (D) Representative fluorescence microscopy images of transfected RDEBK. (E) Cell viability of treated RDEBK. All data were obtained from 3 individual experiments, normalized to untreated RDEBK, and presented as mean ± SD (n = 3). Statistical analysis (one-way ANOVA results with Dunnett's multiple comparison) indicated the difference from PTTA-DATOD. $^{*}p < 0.05$, $^{***}p < 0.001$, and $^{****}p < 0.0001$.

The best condition in both cell lines were used to compare with three commercial gene transfection reagents, including Lipofectamine 3000 (Lipo3000), Xfect, and jetPEI, which were tested at their optimized conditions. For RDEBK transfection, PTTA-DATOD and all commercial reagents showed robust gene delivery capacity (Figures 3A and S6). PTTA-DATOD-treated cells almost had 100% GFP-positive population (Figures 3B and S6) and twice GFP expression level compared with Lipo3000 and Xfect (Figures 3C and 3D). Along with efficient delivery, PTTA-DATOD maintained higher cell viability than all the commercial reagents (Figure 3E). However, all delivery vectors tested here achieved low gene expression only on RDEBF (Figure S7A). Only PTTA-DATOD was able to keep cell viability at more than 70% compared with untreated RDEBF controls, while the other reagents caused a reduction of more than 50% in cell viability (Figure S7B). Therefore, further studies of PTTA-DATOD-mediated gene editing strategy were carried out in RDEBK.

CRISPR plasmid expression in RDEBK

Two plasmids, CRISPR-CMV and CRISPR-CBA (Figure S8), were constructed to deliver the dual sgRNA-guided CRISPR-Cas9 system for human *COL7A1* exon 80 deletion with commonly used cytomegalovirus (CMV) and chick β-actin (CBA) promoters, respectively. Both plasmids were modified from a plasmid named pSpCas9(BB)-2A-GFP (PX458)[38] encoding a GFP-fused *Streptococcus pyogenes* Cas9 (SpCas9) cassette. Therefore, the GFP fluorescence from treated RDEBK can indicate both GFP and SpCas9 nuclease expression. Results from flow cytometry showed that CRISPR-CMV had higher GFP expression level than the CRISPR-CBA plasmid ($p < 0.01$), and PTTA-DATOD/CRISPR-CMV contributed to highest gene expression at W/W of 30 ($p < 0.0001$) (Figures 4A–4C and S9). Robust Cas9 nuclease expression in RDEBK was also observed by immunocytochemistry (ICC) and western blot assays (Figures 4D–4F). Consistent with the GFP pDNA transfection, no obvious effect on cell viability was determined under all the PTTA-DATOD/CRISPR pDNA treatments (Figure 4G).

Targeted deletion of *COL7A1* exon 80

The dual sgRNA-guided CRISPR-Cas9-based *COL7A1* exon 80 deletion strategy used for this work was composed by a pair of sgRNAs targeting intron 79 and intron 80 (listed in Table S3). The genomic DNA from treated RDEBK was extracted and amplified by PCR of

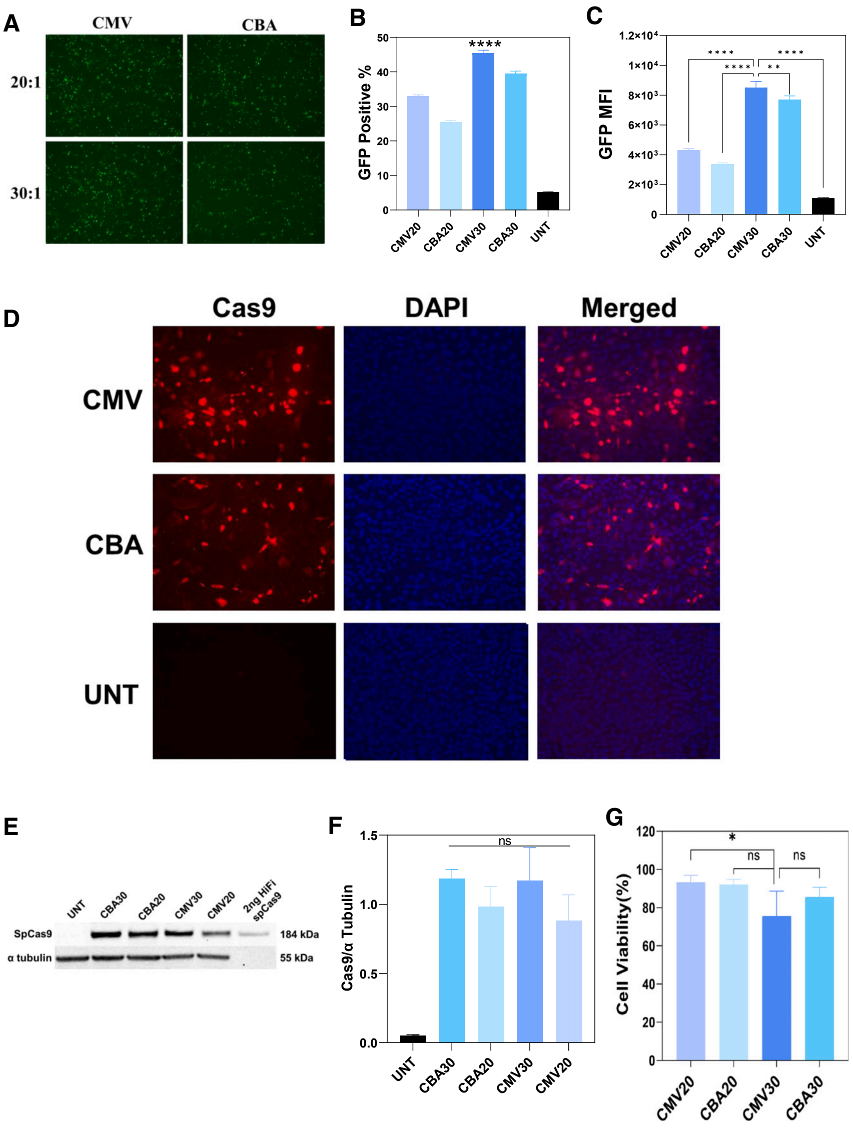

Figure 4. Transfection efficiency analysis of PTTA-DATOD/CRISPR pDNA transfected RDEBK

Cells were transfected by CRISPR-CMV or CRISPR-CBA pDNA at W/W ratios of 20 (CVM20 and CBA20) and 30 (CMV30 and CBA30). At 48 h after transfection, (A) representative fluorescence microscopy images of transfected RDEBK, (B) GFP-positive RDEBK percentage, and (C) mean GFP fluorescence intensity (MFI) were obtained by flow cytometry analysis. (D) Representative immunocytochemistry images of SpCas9 nuclease expression (red) (nucleus stained with DAPI in blue) in RDEBK. (E) SpCas9 nuclease (184 kDa) was detected using western blot, and (F) its expression level was semi-quantitated and normalized with the loading control protein α-tubulin. Band intensity was analyzed by using ImageJ Fiji. (G) Cell viability of transfected RDEBK, normalized to untreated RDEBK. All data were obtained from 3 individual experiments and presented as mean ± SD (n = 3). Statistical analysis (one-way ANOVA results with Dunnett's multiple comparison) results indicated the difference in cells treated with CRISPR-CMV at W/W of 30 (CMV30); ns (no significant difference), $p > 0.05$; *$p < 0.05$, **$p < 0.01$, ***$p < 0.001$, and ****$p < 0.0001$.

a fragment spanning the sgRNA target sites. As shown on the agarose gel image (Figure 5A), shorter bands (263 bp) indicated the successful deletion of exon 80 from the wild-type gene having a band at about 320 bp.

Results of Sanger sequencing of the PCR products revealed precise and efficient targeted gene deletion mediated by PTTA-DATOD/CRISPR pDNA (Figure 5B), although the NHEJ repair between the end of intron 79 and intron 80 is expected to lead to various joining events. CRISPR-CMV contributed to a slightly higher (3% more) exon 80 deletion efficiency than CRISPR-CBA. About 25% of rejoining events corresponded to the fusion between exon 80 flanking cutting sites (56 nt deletion plus a thymine [T] addition), as determined from polyclones treated with both plasmids (Figure 5B). A single cut on intron 79 occurred in 1% of sequences in the polyclonal cell population. It is notable that HPAEs-mediated CRISPR plasmid transfection generated a more specific edited indel spectrum than the indels resulting from electroporation of CRISPR-Cas9 ribonucleoprotein, although the same sgRNAs were used to target the same sites.

COL7A1 transcription and C7 restoration in RDEBK

To further investigate the effect of the NHEJ repair strategy on the following *COL7A1* transcription and splicing, qRT-PCR was performed to detect the mRNA of the corresponding region flanking intron 79 and intron 80. A short band of cDNA amplicons was found on the agarose gel with same size and similar intensity of the genomic DNA (Figure 5C). qRT-PCR results showed that unedited transcripts had similar exon 64 and exon 80 levels, while exon 80 transcription was reduced in the edited samples ($p < 0.001$) (Figure 5D).

Expression of C7 in the edited RDEBK polyclones was assessed using ICC, intracellular immunochemistry staining-based flow cytometry and western blot. The bulk of C7 was detected by ICC in transfected RDEBK polyclones, while no C7 was visualized in the untreated cells (Figure 5E). The C7-expressing RDEBK were quantitatively determined using flow cytometry, the results of which matched the efficiency of genomic editing and transcription (Figures 5F and S10). RDEBK treated using PTTA-DATOD/CRISPR-CMV at W/W of 30 gained highest C7 expression and contributed to 28% C7-positive cells, while the human normal epidermal keratinocytes (HNEK) had 82% C7-positive cells (Figure S10). Western blot of cell extracts

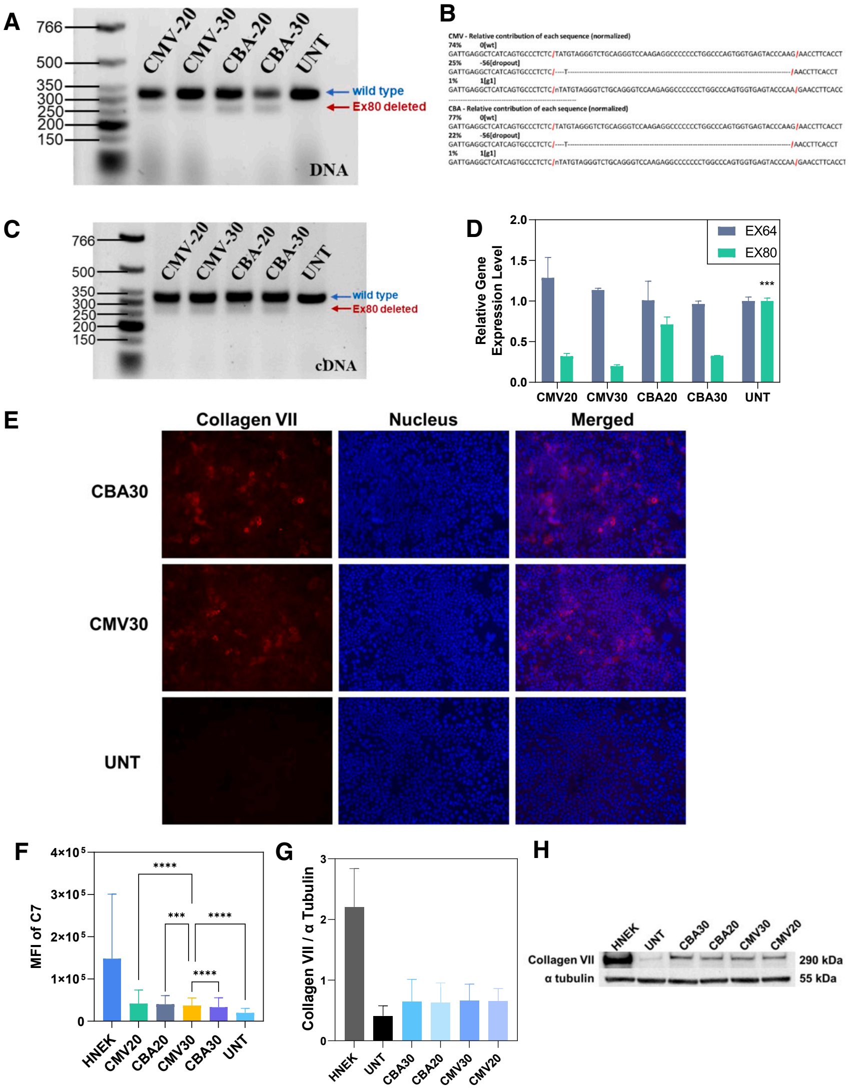

Figure 5. *COL7A1* exon 80 deletion in PTTA-DATOD/CRISPR pDNA transfected RDEBK polyclones

(A) PCR analysis of genomic DNA amplified with primers in exons 78–84, lower band indicates exon 80 deletion compared with unedited wild-type gene upper band. (B) Sanger sequencing analysis and percentage of gene editing events contributing to targeted *COL7A1* exon 80 deletion. (C) RT-PCR analysis of *COL7A1* transcripts amplified with primers in exons 78–84, the lower band indicates transcripts lacking *COL7A1* exon 80 compared with wild-type transcripts. (D) *COL7A1* expression quantification by qRT-PCR using TaqMan probes for *COL7A1* exons 64 and 80. (E) Representative immunocytochemistry images of RDEBK, C7 detected in red, and nucleus stained by DAPI in blue. (F) Mean fluorescence of detected C7 in untreated and transfected RDBEK compared with HNEK was calculated from intracellular immunochemistry staining based flow cytometry analysis. (G) C7 expression levels were semi-quantified and normalized with the loading control protein α-tubulin by band intensity analysis (ImageJ Fiji). (H) Western blot images. All data were obtained from 3 individual experiments and presented as mean ± SD (n = 3). Statistical analysis results (one-way ANOVA results with Dunnett's multiple comparison) indicated the difference from cells treated with PTTA-DATOD/CRISPR-CMV at W/W of 30 (CMV30); ns (no significant difference), $p > 0.05$; *$p < 0.05$, **$p < 0.01$, ***$p < 0.001$, and ****$p < 0.0001$.

also confirmed the C7 expression in the treated RDEBK polyclones. The semi-quantified data show that the PTTA-DATOD/CRISPR pDNA-edited polyclones of RDEBK produced 25%–29% of C7 in HNEK (Figure 5E). The deletion of exon 80 led to loss of 12 amino acids, which was a small size difference and not detectable on the western blot bands (Figure 5H). Overall, these data demonstrate that targeted deletion of exon 80 containing the c.6527dupC mutation can restore the *COL7A1* reading frame and splicing of pre-mRNA and further contribute to C7 expression in RDEB cells.

DISCUSSION

Gene therapy for RDEB has achieved great progress in the past twenty years, with most of the advanced strategies designed as *ex vivo* treatments in order to enhance the efficiency of gene therapy and minimize the safety risk. However, *ex vivo* treatment requires autologous/allogeneic cell culture, gene transduction/editing *in vitro*, three-dimensional (3D) skin equivalent building and transplantation, which faces limitations of laboratory manufacture, invasive surgery, and transplantation rejection risk. Direct delivery of genetic material for topical or systemic treatment can extend the therapeutic application of gene therapies, particularly for patients with severe RDEB who have skin wounds and mucosal fragility. Topical treatment can also be applied during routine wound dressing, minimizing any additional trauma and maximizing convenience to patients.

There have been limited clinical trials focused on topical treatment for RDEB, with most of them being administered through intradermal and intravenous injections or surgical applications. B-VEC, a newly approved gene replacement therapy for topical use, is formulated in a methylcellulose gel and can be applied to the wound site via droplets followed by dressing. No significant increase in anti-HSV antibody was observed in the B-VEC clinical trials (NCT04917887), even after 6 months of administration.[4] However, given the gene replacement nature of B-VEC, long-term administration may be necessary for RDEB patients with extensive wound areas. Consequently, a comprehensive assessment of the safety profile associated with prolonged usage of B-VEC remains imperative. Oleogel-S10 (birch triterpenes) is another promising topical treatment for EB patients. It has finished

phase 3 randomized double-blind phase study with 223 patients and demonstrated its therapeutic effect on accelerated wound healing in EB.[39] The PTW-002 (NCT03605069) is currently undergoing phase 1 and 2 clinical trials as an AON-mediated RNA regulation therapy for RDEB,[6] also specifically designed for topical administration.

The development of topical gene editing therapies for RDEB can bypass the challenge of the large size of *COL7A1* cDNA (~9 kb) in gene replacement treatment and avoid long-term treatment with repeated application. Both homology-directed repair (HDR)-based and NHEJ-based gene editing therapy have been studied as possible therapies for RDEB. HDR-based gene editing requires a donor DNA template, which makes it difficult to achieve high editing efficiency in most cases.[17,18,40–42] Prime editors consisting of CRISPR Cas9 nickase and a RNA template overcome the challenges of traditional HDR-based gene correction, which provides the altered customized sequence simultaneously at the target site with nuclease.[43] However, prime editing is still rarely demonstrated for RDEB treatment.[15] Base editing also offers much more efficient correction than HDR-based gene editing and involves site-specific modification using cytosine base editors or adenine base editors without double-stranded DNA cleavage or exogenous donor template, but it can only be applied to base substitution mutations in *COLA71*.[13,14] NHEJ repairs DNA double-strand breaks by re-joining the two ends of the broken DNA strands. Although NHEJ-based gene editing therapies often results in the introduction of insertion or deletions (indels) at the break site, it provides a potential powerful tool for targeted gene reframing of mutations in *COL7A1* gene.[16,19,32,33,44,45] Here, we implemented an exon deletion strategy to reframe *COL7A1* gene with mutations in exon 80 resulting in premature stop codons and successfully restored bulk C7 production in RDEBK. The feasibility of the exon deletion strategy has been demonstrated *ex vivo* and *in vivo* for RDEB.[16,33,45] Correction of epidermal-dermal adhesion was achieved by electroporation-mediated *ex vivo* and *in vivo* CRISPR-Cas9 ribonucleoprotein delivery.[33,45] However, the high electrical voltage required in those approaches is difficult to apply directly to patients' skin. For *in vivo* topical treatment, Marta et al.[16] reported an adenoviral-based *COL7A1* exon 80 deletion therapy and restored dermal-epidermal adhesion in an RDEB xenograft mouse model. Although adenovirus has high gene transduction ability, the potent immunogenicity is a significant obstacle to its application in gene therapy.[46,47] Marta et al. also detected antibodies against adenovirus in the wounds of immunocompetent mice. Although the antibody level after wound treatment was much lower than those arising after tail vein injection, this issue needs to be further examined as topical treatment for RDEB patients would require higher dosage to be applied to humans in clinical settings. In addition, to address the challenge of limited cargo payload of adenovirus, Marta et al. used *Staphylococcus aureus* Cas9 (SaCas9), as it has a smaller size than the SpCas9 which has been well developed for gene editing.

In this work, we developed HPAEs to deliver CRISPR-Cas9 pDNA for *COL7A1* exon 80 deletion, which has much lower risks of immunogenicity, genotoxicity, and oncogenicity than viral-based gene therapy. PAEs are an evolving class of non-viral vectors that have made significant advancements in the past twenty years. Their larger cargo payload, biodegradability, robust transfection efficiency, and low toxicity make them attractive for gene therapy. Here, we produced HPAEs using a "A2+B4+C2" Michael addition strategy, tested their capacity in DNA encapsulation, release, and intracellular gene delivery. Our study showed that the properties of HPAEs in gene delivery were dependent mainly on the end-capping monomers, as all DAP end-capped HPAEs had stronger pDNA encapsulation ability than DATOD end-capped HPAEs. However, if the interaction between HPAEs and pDNA is too strong, this may impede the pDNA release in the cytosol and result in lower gene expression. Thus, HPAEs with DATOD end-capping contributed to better gene expression level than those with DAP end-capping. Besides, we also noticed the difference between branched units, of which PTTA displayed higher gene expression than other units. The selected HPAEs candidate, PTTA-DATOD, contributed to higher transfection efficiency among all the tested HPAEs. PTTA-DATOD even led to much higher gene expression than Lipo3000 and Xfect in RDEBK and gave the best cell viability of treated cells compared with all tested commercial transfection reagents.

To drive the CRISPR-SpCas9 nuclease expression in RDEBK, two common ubiquitous promoters were compared. CMV enhancer/promoter has often been used to achieve rapid expression, but it is prone to be silenced over time in some tissues. In contrast, the hybrid promoter composed of the CMV enhancer, CBA promoter, and CBA intron usually provides strong and long-term expression in various cells.[48] Through the delivery by PTTA-DATOD, strong expression of SpCas9 nuclease were detected under both promoters. In this study, the two CRISPR pDNAs with CMV or CBA promoters showed no significant difference in gene editing efficiency. Further *in vivo* experiments are required to evaluate the effect of the two promoters.

Our previous work got a targeted exon 80 deletion efficiency of approximately 15% in HEK cells, while achieving a maximum of only 8.2% in RDEBK through CRISPR plasmid delivery.[49] In this study, the PTTA-DATOD-mediated CRISPR-Cas9 pDNA delivery in RDEBK polyclones achieved approximately 25% targeted deletion of *COL7A1* exon 80 *in vitro*, which demonstrated a 3-fold increase in targeted gene editing compared with our previous work. The restoration of C7 production in edited RDEBK polyclones accounted for 29% compared with the C7 production in HNEK. Experimental studies and data analysis of the genotype-phenotype correlations in RDEB patients revealed that about 10% of the C7 found in healthy patients is enough to reduce blistering in RDEB patients and change the severity of the disease to intermediate.[50] The feasibility of PTTA-DATOD-mediated gene therapies has been proven in immortalized RDEBK *in vitro* in this study. In the further work, the efficacy of these non-viral gene therapies is necessary to be evaluated in primary patient keratinocytes, *ex vivo* and *in vivo* RDEB models. The RDEB human skin graft mouse model[33] is a good option, as it can be easily built using RDEB patient skin cells. However, considering that this model employs immunodeficient mice, further safety studies should be

conducted using other animal models, such as wild-type mice and mini pigs, to assess skin irritation and immune response detection.

In terms of gene editing therapy, careful examination of off-target events is crucial for ensuring safety. The dual gRNA guided CRISPR-Cas9 system used in this study was previously reported by Bonafont et al.,[33] who detected off-target variants through next-generation sequencing. They analyzed PCR amplicons covering 244 *in silico*-predicted sites, which did not reveal any off-target events. However, considering the limited delivery efficiency of HPAEs in comparison with electroporation used by Bonafont et al., off-target analysis was not conducted here, as the overall gene editing efficiency was inferior to that achieved through electroporation. Furthermore, the on-target and off-target variants may also be altered when translating *in vitro* transfection to *in vivo* topical treatment that uses GMP grade Cas9 nuclease and sgRNAs. Therefore, further deep sequencing will be performed on DNA samples from *in vivo* RDEB animal models for both on-target and off-target analysis.

To facilitate the clinical translation, it is imperative to further formulate the HPAEs and gene material nanoparticles into a topical medicine. A specialized device, such as a microfluidic device,[51,52] may be required to effectively mix the HPAEs and gene materials and generate uniform nanoparticles on a large scale. Moreover, the application of nanoparticles on wounds for topical administration necessitates the use of a suitable excipient to ensure optimal efficacy and simple operation in clinics.[53,54] An excipient gel or cream would be ideal; however, careful selection and tests are necessary as HPAEs are prone to degradation in aqueous or physiological environments. Furthermore, formulation studies should also consider the stability of the final medicine, its shelf life, and transportation and storage conditions.

In summary, in this work we developed HPAEs with great ability for CRISPR-Cas9 system delivery in epidermal keratinocytes. Our non-viral-based gene editing therapy successfully restored C7 production in RDEB cells harboring the c.6527dupC mutation in *COL7A1 via* CRISPR-Cas9-mediated exon 80 deletion. This non-viral gene editing strategy can be further applied for deleting other *COL7A1* exons containing mutations, and CRISPR-Cas9-based gene editing therapy for other EB subtypes and other genetic diseases.

MATERIALS AND METHODS

Materials

For HPAEs synthesis, the monomers used (BDA, AP, EDA, HMDA, PTTA, DTTA, DAP, and DATOD) were purchased from Merck Life Science (Darmstadt, Germany). DMSO, dimethylformamide (DMF), diethyl ether, and deuterated chloroform (CDCl3) were also purchased from Merck Life Science.

HEK cells were ordered from American Type Culture Collection (Manassas, VA). HNEK were obtained from PromoCell (Heidelberg, Germany). Immortalized RDEBK and RDEBF were kindly provided by Dr. F. Larcher (Centro de Investigaciones Energéticas, Medioambientales y Tecnológicas [CIEMAT], Madrid, Spain). Hank's balanced salt solution (HBSS), 3 M sodium acetate (SA), FluoroShield with DAPI, and DMEM 6249 were purchased from Merck Life Science.

jetPEI was ordered from Polyplus Transfection (Illkirch-Graffenstaden, France), and Xfect was purchased from Takara Bio (Saint-Germain-en-Laye, France). Quant-iT PicoGreen dsDNA Assay Kits, alamarBlue, Lipo3000, fetal bovine serum (FBS), penicillin/streptomycin, bicinchoninic acid (BCA) Protein Assay Kit, 3%–8% gradient Tris-acetate gels, and Pierce ECL Western Blotting Substrate were ordered from Thermo Fisher Scientific (Waltham, MA). gWiz-GFP plasmids were purchased from Aldevron (Fargo, ND). cOmplete Mini Protease Inhibitor Cocktail was ordered from Roche (Basel, Switzerland). For C7 intracellular staining flow cytometry, fixation buffer, intracellular staining permeabilization wash buffer and stain buffer were purchased from BioLegend. Rabbit anti-C7 antibody was kindly provided by Dr. Alexander Nyström (University of Freiburg, Freiburg, Germany). Rabbit monoclonal anti-spCas9 primary antibody (EPR18991) was ordered from Abcam (Cambridge, UK). Rabbit anti-α-tubulin antibody and anti-rabbit IgG (horseradish peroxidase [HRP]-linked antibody; #7074) were ordered from Cell Signaling Technology (Danvers, MA). Goat anti-rabbit IgG (H&L)-Alexa Fluor 568 was purchased from Thermo Fisher Scientific. For the qRT-PCR assays, the RNeasy Mini Kit was ordered from QIAGEN, SuperScript IV First-Strand Synthesis kit, TaqMan Real-Time PCR Master Mixes, and TaqMan probes were purchased from Thermo Fisher Scientific.

HPAEs synthesis

BDA, branching monomer (EDA, HMDA, PTTA, DTTA), and S5 were mixed in a flask, with DMSO as the solvent, to achieve a monomer concentration of 30% (w/w). The reaction was performed at 90°C until the ideal Mw was achieved. The reaction was stopped by removing the reaction flask from heat and cooled with ice. End-capping monomer (DAP or DATOD) was added into the flask with DMSO (a final monomer concentration of 10% W/W) to react with the residual acrylate for 48 h at room temperature (RT). Afterward, the reaction mixture was precipitated into excess amount of diethyl ether 4 or 5 times to remove the monomers and oligomers. The resulting viscous liquid was dried with vacuum oven at RT to produce the final polymer.

^{1}H-NMR characterization

Eight to 12 mg of the polymer samples were dissolved in 700 μL $CDCl_3$. The ^{1}H-NMR results were collected on a Varian 400 MHz spectrometer (Edinburgh, UK). The chloroform ($CHCl_3$) peak (7.26) was used as the reference peak.

Gel permeation chromatography characterization

The number average molar mass (Mn), Mw, and PDI of the polymers were tested by an Agilent 1260 Infinity gel permeation chromatograph (Agilent Technologies, Santa Clara, CA) equipped with a triple detector including refractive index detector, viscometer detector, and

dual-angle light scattering detector (LS 15° and LS 90°). To test the Mw during the polymerization process, 50 μL of the reaction solution was sampled and diluted with 1 mL DMF, followed by filtration through a 0.2 μm filter. The gel permeation chromatograph was running DMF with 0.1% lithium bromide (LiBr) as the mobile phase at a flow rate of 1 mL/min at 60°C. The gel permeation chromatograph was calibrated with poly(methyl methacrylate) (PMMA) standards.

HPAEs and DNA nanoparticle complexation

The polymer was dissolved in DMSO to 100 μg/μL stock solution and stored at −20°C for the following studies. All the pDNA was stored in Tris-EDTA (TE) buffer at concentration of 1 μg/μL. For HPAEs/pDNA complex preparation, each polymer and pDNA stock solution were dissolved in 25 mM SA to equal volume according to the desired HPAEs to pDNA weight ratios (W/W). Then, the HPAEs/SA solution was added to the pDNA/SA solution, mixed well by pipetting, and incubated for 5–10 min at RT to form HPAEs/pDNA complexes for subsequent studies.

DNA binding assays

Quant-iT PicoGreen dsDNA Assay Kits were used to quantify the DNA binding capacity of each polymer. HPAEs/pDNA complexes were prepared as described above, added to a black 96-well plate with equal volume (100 μL) of PicoGreen working solution and incubated for 5 min protected from light at RT. Fluorescence was measured using a SpectraMax M3 plate reader (Molecular Devices, San Jose, CA). DNA BE was calculated as follows:

$$BE = \left(F_{DNA} - F_{Sample}\right) / \left(F_{DNA} - F_{Blank}\right) \times 100\%. \qquad \text{(Equation 1)}$$

F_{DNA} was the fluorescence from free pDNA without polymer, F_{Sample} was the fluorescence from complexes at a given weight ratio between polymer to pDNA, and F_{Blank} was the fluorescence from PicoGreen working solution with the buffer used for HPAEs/pDNA formulation.

Agarose gel retardation was also used to confirm DNA encapsulation ability of HPAEs. Briefly, 10 μL of HPAEs/pDNA complexes were loaded in a 1% agarose gel, and the electrophoresis was run at 110V for 60 min. DNA bands were observed using the iBright CL750 Imaging System (Thermo Fisher Scientific).

HPAEs/pDNA complex characterization

The sizes and zeta potential of complexes formulated by HPAEs and GFP pDNA at a W/W of 20 were measured using Zetasizer Pro (Malvern Panalytical Ltd., Cambridge, UK). The complexes were prepared as described above and diluted to 1 mL of distilled water for measurement. The temperature of the samples was controlled at 25°C.

Cell culture and transfection

Immortalized RDEBF and HEK cells were cultured in DMEM with 10% FBS and 1% penicillin/streptomycin. HNEK and immortalized RDEBK were cultured using standard cell culture techniques in keratinocyte growth complete FAD medium (KCa) as described by Bonafont et al.[33] and incubated at 37°C and 5% CO_2 in a humidified incubator. HEK cells were seeded at cell density of 62,500 cells/cm^2 for transfection, while RDEBK and HNEK cells were seeded at 31,250 cells/cm^2 and RDEBF were seeded at 21,875 cells/cm^2.

Twenty-four hours after cell seeding, fresh HPAEs/pDNA complexes were prepared as described above and added to full cell culture media at 10% concentration (v/v) to replace the old media in the cell culture plates. All the treated cells were incubated at 37°C and 5% CO_2 with HPAEs/pDNA complexes without changing media until examining transfection efficiency. The transfection with Lipo3000 was carried out according to its commercial protocol at a dose of 0.3 μL Lipo3000 reagent per 100 ng DNA. Xfect and jetPEI were used as described in their manuals as appropriate for the selected cell culture vessels.

Cell viability assay

Cell viability was assessed 48 h post-transfection. Medium was removed, and cells were washed with 100 μL of HBSS per well. Then, 100 μL alamarBlue working solution (10% alamarBlue in HBSS) was added to each well and incubated for 1 h under normal cell culture conditions as previously described. Absorbance was recorded at 570 nm and 600 nm by SpectraMax M3 multi-plate reader (Molecular Devices, San Jose, CA). Untreated cells were used to normalize fluorescence values and set as 100% viable. Wells containing only alamarBlue reagent were subtracted as background prior to obtaining the percentage of cell viability, calculated as follows:

$$\frac{Absorbance\ of\ treated\ cells - background}{Absorbance\ of\ untreated\ cells - background} \times 100\%. \qquad \text{(Equation 2)}$$

GFP expression analysis

The expression of GFP protein from HPAEs/GFP pDNA was visualized using an Olympus IX81 fluorescence microscope (Olympus Life Science, Tokyo, Japan) and quantified using CytoFLEX Flow Cytometer (Beckman Coulter Life Sciences, Indianapolis, IN) 48 h post-transfection. For fluorescence microscope observation, the cell culture media was firstly removed from cells and cells were washed once with HBSS. Images were randomly taken from the center of each well (one image per well). Each condition had three replicates. The fluorescent images were further analyzed using ImageJ Fiji software to semi-quantify the fluorescent intensity. Alternatively, the transfected cells were detached from the plate by trypsin treatment and washed twice with HBSS. After centrifugation, cells were resuspended in 400 μL HBSS for flow cytometry analysis at Ex/Em = 488/525 nm.

ICC for SpCas9 nuclease and C7

Cells were seeded on 9 mm glass coverslips in a 48-well plate and treated with HPAEs/CRISPR pDNA. Twenty-four hours after transfection, cell culture media was removed, and coverslips were washed three times with ice-cold PBS. Cells were fixed with ice-cold

acetone/methanol (v/v = 1:1) for 20 min at −20°C. Samples were washed three times with PBS and then were incubated in 3% bovine serum albumin for 20 min at RT. For ICC of SpCas9 nuclease, a rabbit monoclonal anti-SpCas9 primary antibody (EPR18991) was used to incubate samples at a dilution of 1:100 in 0.1% BSA/PBS solution overnight at 4°C. After washing three times, coverslips were incubated with goat anti-rabbit IgG (H&L)-Alexa Fluor 568 at 1:800 dilution for 1 h at RT, mounted on microscope slides with FluoroShield mounting medium with DAPI, and imaged using an Olympus IX83 microscope. For C7 detection, a rabbit anti-C7 primary antibody (kindly provided by Dr. Alexander Nyström)[55] at a dilution of 1:5,000 was used to incubate the cells, and then incubated with goat anti-rabbit IgG (H&L)-Alexa Fluor 568 at 1:800 dilution.

Western blotting of SpCas9 nuclease and C7

Cell lysates were harvested using radioimmunoprecipitation assay (RIPA) buffer containing cOmplete Mini Protease Inhibitor Cocktail after transfection for 72 h. The lysates were frozen at −80°C overnight and then centrifuged for 10 min at 14800 rpm at 4°C for protein supernatant collection. Amount of total protein in each sample was quantified using the BCA Protein Assay Kit and separated in 3%–8% gradient Tris-acetate gels to be later transferred to a nitrocellulose membrane by electrophoresis at 90 V for 90 min. To detect SpCas9 nuclease, the membrane was incubated with rabbit monoclonal anti-SpCas9 antibody at 1:20,000 diluted in milk blocking solution overnight at 4°C. Rabbit anti-C7 antibody was used at a dilution of 1:5,000 to detect C7 on the membrane. As loading control, anti-human α-tubulin antibody was used at a dilution of 1:1,000. The secondary antibodies for the corresponding primary antibodies, anti-rabbit IgG (HRP-linked) antibody, was used at 1:2,000 dilution. Protein bands were developed using the Pierce ECL Western Blotting Substrate and the iBright CL750 Imaging System. Semi-quantitative analysis of C7 bands was performed using ImageJ Fiji software. Both SpCas9 nuclease and C7 bands were normalized to the loading control α-tubulin.

Intracellular staining flow cytometry

Intracellular staining of flow cytometry of C7 assay was performed from a modified commercial protocol provided by BioLegend. At 72 h post-transfection, cells were detached from the plate, and fixed in Fixation Buffer at RT for 20 min in the dark. Afterward, cells were permeabilized by resuspension in Intracellular Staining Permeabilization Wash Buffer and blocked in Stain Buffer for 15 min. The primary anti-C7 antibody was added at a dilution of 1:1,000 to be later incubated with the goat anti-rabbit IgG (H&L)-Alexa Fluor 568 at a dilution of 1:200. Cells were tested using a CytoFLEX Flow Cytometer. Data analysis was performed using the CytExpert software (Beckman Coulter Life Sciences).

DNA isolation and PCR

Genomic DNA of cells were extracted 72 h after transfection using lysis buffer (Tris [pH 8] 100 mM, EDTA 5 mM, SDS 0.2%, NaCl 200 mM, and 1 mg/mL Proteinase K) and ethanol precipitation. The target gene was amplified with the primers (forward primer: GGGGTCAGGACGGCAAC; reverse primer: CAGCTCCAGTAGG TCCAGTCAG) using DNA polymerase master mix. The PCR products were separated by electrophoresis and visualized on a 1.8% agarose gel. The PCR products also were sent to Eurofins MWG Operon Inc. (Luxembourg, Luxembourg) for Sanger sequencing. Inference of CRISPR Edits (ICE) Analysis Tool (Synthego, Redwood City, CA) was used to confirm editing events and to track formation of indels in a pooled cell population.

RNA isolation and qRT-PCR

The RNeasy Mini Kit was used for total RNA extraction from cells at 72 h post-transfection. Total RNA (0.1 μg) from each sample was used to synthesize the first-strand cDNA with the primer Oligo (dT)20 (50 μM) according to the protocol of SuperScript IV First-Strand Synthesis kit. For qPCR analysis, 1 μL of the final cDNA product was added to 9 μL of reaction mix (0.5 μL targeted gene TaqMan primer, 0.5 μL human glyceraldehyde 3-phosphate dehydrogenase [GAPDH] endogenous control TaqMan primers (Table S3), 5 μL TaqMan PCR mix, and 3 μL RNase-free water), which were loaded on a 384-well plate and tested using a QuantStudio 7 Flex System (Thermo Fisher Scientific). Each sample was measured in triplicate. For quantification of *COL7A1* exon gene transcription, human GAPDH was used as the endogenous control. Results were analyzed using QuantStudio Real-Time PCR software (Thermo Fisher Scientific).

Statistical analysis

All data are represented as mean ± SD, normally performing a minimum of three independent experiments. Analysis was carried out using one-way ANOVA with Dunnett's multiple-comparison tests using Prism 8.0 (GraphPad, San Diego, CA). p values of less than 0.05 were considered significant (*$p < 0.05$, **$p < 0.01$, ***$p < 0.001$, and ****$p < 0.0001$). Statistical significance was reported in the figure legends.

DATA AND CODE AVAILABILITY

The authors confirm that the data supporting the findings of this study are available within the article and its supplemental information. Additional sequencing data are available upon request.

SUPPLEMENTAL INFORMATION

Supplemental information can be found online at https://doi.org/10.1016/j.omtm.2023.101134.

ACKNOWLEDGMENTS

This research was supported by the Chinese Government–China Scholarship Council (CSC202008300001) and Science Foundation Ireland funded project 19/FFP/6522-Frontiers for the Future Award (Republic of Ireland). The authors would like to thank Dr. Alfonso Blanco, director of the Flow Cytometry Core facility in the University College Dublin (UCD) Conway Institute for his assistance in guiding the flow cytometry work, the core technologist at the UCD Conway Genomics Core for her assistance in guiding the qRT-PCR work, Dr. Alexander Nyström (University of Freiburg, Freiburg, Germany)

for the donation of the rabbit anti-C7 antibody used in this project, and Dr. Fernando Larcher (CIEMAT, Madrid, Spain) for the donation of the immortalized RDEBK and RDEBF used in this project. The authors would also like to thank Ruth Foley her assistance in revising English grammar and style.

AUTHOR CONTRIBUTIONS

Conceptualization, methodology, investigation, writing – original draft, and funding acquisition, Xianqing W.; writing – review & editing, Xi W.; investigation, Y.L., S.A., B.Q., A.B., and Z.H.; supervision, writing – review & editing, and funding acquisition, W.W.; supervision, writing – review & editing, and funding acquisition, I.L.-S.

DECLARATION OF INTERESTS

The authors declare no competing interests. W.W. is the founder of Branca Bunús, a UCD startup company that manufactures branched polymers for gene delivery and in which Xianqing W. is involved in collaborative research projects.

REFERENCES

1. Bardhan, A., Bruckner-Tuderman, L., Chapple, I.L.C., Fine, J.-D., Harper, N., Has, C., Magin, T.M., Marinkovich, M.P., Marshall, J.F., McGrath, J.A., et al. (2020). Epidermolysis bullosa. Nat. Rev. Dis. Prim. *6*, 78. https://doi.org/10.1038/s41572-020-0210-0.
2. Togo, C.C.G., Zidorio, A.P.C., Gonçalves, V.S.S., Hubbard, L., de Carvalho, K.M.B., and Dutra, E.S. (2020). Quality of life in people with epidermolysis bullosa: a systematic review. Qual. Life Res. *29*, 1731–1745. https://doi.org/10.1007/s11136-020-02495-5.
3. Tang, J.Y., Marinkovich, M.P., Lucas, E., Gorell, E., Chiou, A., Lu, Y., Gillon, J., Patel, D., and Rudin, D. (2021). A systematic literature review of the disease burden in patients with recessive dystrophic epidermolysis bullosa. Orphanet J. Rare Dis. *16*, 175. https://doi.org/10.1186/s13023-021-01811-7.
4. Gurevich, I., Agarwal, P., Zhang, P., Dolorito, J.A., Oliver, S., Liu, H., Reitze, N., Sarma, N., Bagci, I.S., Sridhar, K., et al. (2022). In vivo topical gene therapy for recessive dystrophic epidermolysis bullosa: a phase 1 and 2 trial. Nat. Med. *28*, 780–788. https://doi.org/10.1038/s41591-022-01737-y.
5. Pendaries, V., Gasc, G., Titeux, M., Tonasso, L., Mejía, J.E., and Hovnanian, A. (2012). siRNA-Mediated Allele-Specific Inhibition of Mutant Type VII Collagen in Dominant Dystrophic Epidermolysis Bullosa. J. Invest. Dermatol. *132*, 1741–1743. https://doi.org/10.1038/jid.2012.11.
6. Bornert, O., Hogervorst, M., Nauroy, P., Bischof, J., Swildens, J., Athanasiou, I., Tufa, S.F., Keene, D.R., Kiritsi, D., Hainzl, S., et al. (2021). QR-313, an Antisense Oligonucleotide, Shows Therapeutic Efficacy for Treatment of Dominant and Recessive Dystrophic Epidermolysis Bullosa: A Preclinical Study. J. Invest. Dermatol. *141*, 883–893.e6. https://doi.org/10.1016/j.jid.2020.08.018.
7. Turczynski, S., Titeux, M., Tonasso, L., Décha, A., Ishida-Yamamoto, A., and Hovnanian, A. (2016). Targeted Exon Skipping Restores Type VII Collagen Expression and Anchoring Fibril Formation in an In Vivo RDEB Model. J. Invest. Dermatol. *136*, 2387–2395. https://doi.org/10.1016/j.jid.2016.07.029.
8. Tockner, B., Kocher, T., Hainzl, S., Reichelt, J., Bauer, J.W., Koller, U., and Murauer, E.M. (2016). Construction and validation of an RNA trans-splicing molecule suitable to repair a large number of COL7A1 mutations. Gene Ther. *23*, 775–784. https://doi.org/10.1038/gt.2016.57.
9. Dallinger, G., Puttaraju, M., Mitchell, L.G., Yancey, K.B., Yee, C., Klausegger, A., Hintner, H., and Bauer, J.W. (2003). Development of spliceosome-mediated RNA trans-splicing (SMaRT^{TM}) for the correction of inherited skin diseases. Exp. Dermatol. *12*, 37–46. https://doi.org/10.1034/j.1600-0625.2003.120105.x.
10. Izmiryan, A., Danos, O., and Hovnanian, A. (2016). Meganuclease-Mediated COL7A1 Gene Correction for Recessive Dystrophic Epidermolysis Bullosa. J. Invest. Dermatol. *136*, 872–875. https://doi.org/10.1016/j.jid.2015.11.028.
11. Osborn, M.J., Starker, C.G., McElroy, A.N., Webber, B.R., Riddle, M.J., Xia, L., DeFeo, A.P., Gabriel, R., Schmidt, M., Von Kalle, C., et al. (2013). TALEN-based Gene Correction for Epidermolysis Bullosa. Mol. Ther. *21*, 1151–1159. https://doi.org/10.1038/mt.2013.56.
12. Chamorro, C., Mencía, A., Almarza, D., Duarte, B., Büning, H., Sallach, J., Hausser, I., Del Río, M., Larcher, F., and Murillas, R. (2016). Gene Editing for the Efficient Correction of a Recurrent COL7A1 Mutation in Recessive Dystrophic Epidermolysis Bullosa Keratinocytes. Mol. Ther. Nucleic Acids *5*, e307. https://doi.org/10.1038/mtna.2016.19.
13. Naso, G., Gkazi, S.A., Georgiadis, C., Jayarajan, V., Jacków, J., Fleck, R., Allison, L., Ogunbiyi, O.K., McGrath, J.A., Ilic, D., et al. (2023). Cytosine deaminase base editing to restore COL7A1 in dystrophic epidermolysis bullosa human:murine skin model. JID Innov. *3*, 100191. https://doi.org/10.1016/j.xjidi.2023.100191.
14. Sheriff, A., Guri, I., Zebrowska, P., Llopis-Hernandez, V., Brooks, I.R., Tekkela, S., Subramaniam, K., Gebrezgabher, R., Naso, G., Petrova, A., et al. (2022). ABE8e adenine base editor precisely and efficiently corrects a recurrent COL7A1 nonsense mutation. Sci. Rep. *12*, 19643. https://doi.org/10.1038/s41598-022-24184-8.
15. Hong, S.-A., Kim, S.-E., Lee, A.-Y., Hwang, G.-H., Kim, J.H., Iwata, H., Kim, S.-C., Bae, S., and Lee, S.E. (2022). Therapeutic base editing and prime editing of COL7A1 mutations in recessive dystrophic epidermolysis bullosa. Mol. Ther. *30*, 2664–2679. https://doi.org/10.1016/j.ymthe.2022.06.005.
16. García, M., Bonafont, J., Martínez-Palacios, J., Xu, R., Turchiano, G., Svensson, S., Thrasher, A.J., Larcher, F., Del Rio, M., Hernández-Alcoceba, R., et al. (2022). Preclinical model for phenotypic correction of dystrophic epidermolysis bullosa by in vivo CRISPR-Cas9 delivery using adenoviral vectors. Mol. Ther. Methods Clin. Dev. *27*, 96–108. https://doi.org/10.1016/j.omtm.2022.09.005.
17. Kocher, T., Bischof, J., Haas, S.A., March, O.P., Liemberger, B., Hainzl, S., Illmer, J., Hoog, A., Muigg, K., Binder, H.-M., et al. (2021). A non-viral and selection-free COL7A1 HDR approach with improved safety profile for dystrophic epidermolysis bullosa. Mol. Ther. Nucleic Acids *25*, 237–250. https://doi.org/10.1016/j.omtn.2021.05.015.
18. Bonafont, J., Mencía, A., Chacón-Solano, E., Srifa, W., Vaidyanathan, S., Romano, R., Garcia, M., Hervás-Salcedo, R., Ugalde, L., Duarte, B., et al. (2021). Correction of recessive dystrophic epidermolysis bullosa by homology-directed repair-mediated genome editing. Mol. Ther. *29*, 2008–2018. https://doi.org/10.1016/j.ymthe.2021.02.019.
19. Kocher, T., March, O.P., Bischof, J., Liemberger, B., Hainzl, S., Klausegger, A., Hoog, A., Strunk, D., Bauer, J.W., and Koller, U. (2020). Predictable CRISPR/Cas9-Mediated COL7A1 Reframing for Dystrophic Epidermolysis Bullosa. J. Invest. Dermatol. *140*, 1985–1993.e5. https://doi.org/10.1016/j.jid.2020.02.012.
20. Stewart, M.P., Langer, R., and Jensen, K.F. (2018). Intracellular Delivery by Membrane Disruption: Mechanisms, Strategies, and Concepts. Chem. Rev. *118*, 7409–7531. https://doi.org/10.1021/acs.chemrev.7b00678.
21. Shi, J., Ma, Y., Zhu, J., Chen, Y., Sun, Y., Yao, Y., Yang, Z., and Xie, J. (2018). A Review on Electroporation-Based Intracellular Delivery. Molecules *23*, 3044. https://doi.org/10.3390/molecules23113044.
22. Yang, N.-S., and Sun, W.H. (1995). Gene gun and other non-viral approaches for cancer gene therapy. Nat. Med. *1*, 481–483. https://doi.org/10.1038/nm0595-481.
23. Xu, W. (2019). Microinjection and Micromanipulation: A Historical Perspective. In Microinjection Methods in Molecular Biology, C. Liu and Y. Du, eds. (Springer New York), pp. 1–16. https://doi.org/10.1007/978-1-4939-8831-0_1.
24. Rouatbi, N., McGlynn, T., and Al-Jamal, K.T. (2022). Pre-clinical non-viral vectors exploited for in vivo CRISPR/Cas9 gene editing: an overview. Biomater. Sci. *10*, 3410–3432. https://doi.org/10.1039/D1BM01452H.
25. Zu, H., and Gao, D. (2021). Non-viral Vectors in Gene Therapy: Recent Development, Challenges, and Prospects. AAPS J. *23*, 78. https://doi.org/10.1208/s12248-021-00608-7.
26. Akinc, A., Lynn, D.M., Anderson, D.G., and Langer, R. (2003). Parallel Synthesis and Biophysical Characterization of a Degradable Polymer Library for Gene Delivery. J. Am. Chem. Soc. *125*, 5316–5323. https://doi.org/10.1021/ja034429c.
27. Wei, J., Zhu, L., Lu, Q., Li, G., Zhou, Y., Yang, Y., and Zhang, L. (2023). Recent progress and applications of poly(beta amino esters)-based biomaterials. J. Contr. Release *354*, 337–353. https://doi.org/10.1016/j.jconrel.2023.01.002.

28. Zeng, M., Xu, Q., Zhou, D.A.S., Alshehri, F., Lara-Sáez, I., Zheng, Y., Li, M., Wang, W., and Wang, W. (2021). Highly branched poly(β-amino ester)s for gene delivery in hereditary skin diseases. Adv. Drug Deliv. Rev. *176*, 113842. https://doi.org/10.1016/j.addr.2021.113842.

29. Zhou, D., Cutlar, L., Gao, Y., Wang, W., O'Keeffe-Ahern, J., McMahon, S., Duarte, B., Larcher, F., Rodriguez, B.J., Greiser, U., and Wang, W. (2016). The transition from linear to highly branched poly(β-amino ester)s: Branching matters for gene delivery. Sci. Adv. *2*, e1600102. https://doi.org/10.1126/sciadv.1600102.

30. Zhou, D., Gao, Y., Aied, A., Cutlar, L., Igoucheva, O., Newland, B., Alexeeve, V., Greiser, U., Uitto, J., and Wang, W. (2016). Highly branched poly(β-amino ester)s for skin gene therapy. J. Control Release. *244*, 336–346. https://doi.org/10.1016/j.jconrel.2016.06.014.

31. Sanchez-Jimeno, C., Cuadrado-Corrales, N., Aller, E., García, M., Escámez, M.J., Illera, N., Trujillo-Tiebas, M.J., Ayuso, C., Millán, J.M., and Del Río, M. (2013). Recessive dystrophic epidermolysis bullosa: the origin of the c.6527insC mutation in the Spanish population: Correspondence. Br. J. Dermatol. *168*, 226–229. https://doi.org/10.1111/j.1365-2133.2012.11128.x.

32. Mencía, Á., Chamorro, C., Bonafont, J., Duarte, B., Holguin, A., Illera, N., Llames, S.G., Escámez, M.J., Hausser, I., Del Río, M., et al. (2018). Deletion of a Pathogenic Mutation-Containing Exon of COL7A1 Allows Clonal Gene Editing Correction of RDEB Patient Epidermal Stem Cells. Mol. Ther. Nucleic Acids *11*, 68–78. https://doi.org/10.1016/j.omtn.2018.01.009.

33. Bonafont, J., Mencía, Á., García, M., Torres, R., Rodríguez, S., Carretero, M., Chacón-Solano, E., Modamio-Høybjør, S., Marinas, L., León, C., et al. (2019). Clinically Relevant Correction of Recessive Dystrophic Epidermolysis Bullosa by Dual sgRNA CRISPR/Cas9-Mediated Gene Editing. Mol. Ther. *27*, 986–998. https://doi.org/10.1016/j.ymthe.2019.03.007.

34. Bremer, J., Bornert, O., Nyström, A., Gostynski, A., Jonkman, M.F., Aartsma-Rus, A., van den Akker, P.C., and Pasmooij, A.M. (2016). Antisense Oligonucleotide-mediated Exon Skipping as a Systemic Therapeutic Approach for Recessive Dystrophic Epidermolysis Bullosa. Mol. Ther. Nucleic Acids *5*, e379. https://doi.org/10.1038/mtna.2016.87.

35. Bornert, O., Kühl, T., Bremer, J., van den Akker, P.C., Pasmooij, A.M., and Nyström, A. (2016). Analysis of the functional consequences of targeted exon deletion in COL7A1 reveals prospects for dystrophic epidermolysis bullosa therapy. Mol. Ther. *24*, 1302–1311. https://doi.org/10.1038/mt.2016.92.

36. Chen, M., O'Toole, E.A., Muellenhoff, M., Medina, E., Kasahara, N., and Woodley, D.T. (2000). Development and Characterization of A Recombinant Truncated Type VII Collagen "Minigene. J. Biol. Chem. *275*, 24429–24435. https://doi.org/10.1074/jbc.M003440200.

37. Zeng, M., Alshehri, F., Zhou, D., Lara-Sáez, I., Wang, X., Li, X.A.S., Xu, Q., Zhang, J., Wang, W., and Wang, W. (2019). Efficient and Robust Highly Branched Poly(β-amino ester)/Minicircle *COL7A1* Polymeric Nanoparticles for Gene Delivery to Recessive Dystrophic Epidermolysis Bullosa Keratinocytes. ACS Appl. Mater. Interfaces *11*, 30661–30672. https://doi.org/10.1021/acsami.9b13135.

38. Ran, F.A., Hsu, P.D., Wright, J., Agarwala, V., Scott, D.A., and Zhang, F. (2013). Genome engineering using the CRISPR-Cas9 system. Nat. Protoc. *8*, 2281–2308. https://doi.org/10.1038/nprot.2013.143.

39. Kern, J.S., Sprecher, E., Fernandez, M.F., Schauer, F., Bodemer, C., Cunningham, T., Löwe, S., Davis, C., Sumeray, M., Bruckner, A.L., et al. (2023). Efficacy and safety of Oleogel-S10 (birch triterpenes) for epidermolysis bullosa: results from the phase III randomized double-blind phase of the EASE study. Br. J. Dermatol. *188*, 12–21. https://doi.org/10.1093/bjd/ljac001.

40. Izmiryan, A., Ganier, C., Bovolenta, M., Schmitt, A., Mavilio, F., and Hovnanian, A. (2018). Ex Vivo COL7A1 Correction for Recessive Dystrophic Epidermolysis Bullosa Using CRISPR/Cas9 and Homology-Directed Repair. Mol. Ther. Nucleic Acids *12*, 554–567. https://doi.org/10.1016/j.omtn.2018.06.008.

41. Jacków, J., Guo, Z., Hansen, C., Abaci, H.E., Doucet, Y.S., Shin, J.U., Hayashi, R., DeLorenzo, D., Kabata, Y., Shinkuma, S., et al. (2019). CRISPR/Cas9-based targeted genome editing for correction of recessive dystrophic epidermolysis bullosa using iPS cells. Proc. Natl. Acad. Sci. USA *116*, 26846–26852. https://doi.org/10.1073/pnas.1907081116.

42. Kocher, T., Wagner, R.N., Klausegger, A., Guttmann-Gruber, C., Hainzl, S., Bauer, J.W., Reichelt, J., and Koller, U. (2019). Improved Double-Nicking Strategies for COL7A1-Editing by Homologous Recombination. Mol. Ther. Nucleic Acids *18*, 496–507. https://doi.org/10.1016/j.omtn.2019.09.011.

43. Marzec, M., Brąszewska-Zalewska, A., and Hensel, G. (2020). Prime Editing: A New Way for Genome Editing. Trends Cell Biol. *30*, 257–259. https://doi.org/10.1016/j.tcb.2020.01.004.

44. Takashima, S., Shinkuma, S., Fujita, Y., Nomura, T., Ujiie, H., Natsuga, K., Iwata, H., Nakamura, H., Vorobyev, A., Abe, R., and Shimizu, H. (2019). Efficient Gene Reframing Therapy for Recessive Dystrophic Epidermolysis Bullosa with CRISPR/Cas9. J. Invest. Dermatol. *139*, 1711–1721.e4. https://doi.org/10.1016/j.jid.2019.02.015.

45. Wu, W., Lu, Z., Li, F., Wang, W., Qian, N., Duan, J., Zhang, Y., Wang, F., and Chen, T. (2017). Efficient in vivo gene editing using ribonucleoproteins in skin stem cells of recessive dystrophic epidermolysis bullosa mouse model. Proc. Natl. Acad. Sci. USA *114*, 1660–1665. https://doi.org/10.1073/pnas.1614775114.

46. Zaiss, A.K., Machado, H.B., and Herschman, H.R. (2009). The influence of innate and pre-existing immunity on adenovirus therapy. J. Cell. Biochem. *108*, 778–790. https://doi.org/10.1002/jcb.22328.

47. Shirley, J.L., de Jong, Y.P., Terhorst, C., and Herzog, R.W. (2020). Immune Responses to Viral Gene Therapy Vectors. Mol. Ther. *28*, 709–722. https://doi.org/10.1016/j.ymthe.2020.01.001.

48. Gray, S.J., Foti, S.B., Schwartz, J.W., Bachaboina, L., Taylor-Blake, B., Coleman, J., Ehlers, M.D., Zylka, M.J., McCown, T.J., and Samulski, R.J. (2011). Optimizing Promoters for Recombinant Adeno-Associated Virus-Mediated Gene Expression in the Peripheral and Central Nervous System Using Self-Complementary Vectors. Hum. Gene Ther. *22*, 1143–1153. https://doi.org/10.1089/hum.2010.245.

49. O'Keeffe Ahern, J., Lara-Sáez, I., Zhou, D., Murillas, R., Bonafont, J., Mencía, Á., García, M., Manzanares, D., Lynch, J., Foley, R., et al. (2022). Non-viral delivery of CRISPR–Cas9 complexes for targeted gene editing via a polymer delivery system. Gene Ther. *29*, 157–170. https://doi.org/10.1038/s41434-021-00282-6.

50. Uitto, J., Has, C., Vahidnezhad, H., Youssefian, L., and Bruckner-Tuderman, L. (2017). Molecular pathology of the basement membrane zone in heritable blistering diseases. Matrix Biol. *57–58*, 76–85. https://doi.org/10.1016/j.matbio.2016.07.009.

51. Liu, Y., Yang, G., Hui, Y., Ranaweera, S., and Zhao, C.X. (2022). Microfluidic Nanoparticles for Drug Delivery. Small *18*, 2106580. https://doi.org/10.1002/smll.202106580.

52. Prakash, G., Shokr, A., Willemen, N., Bashir, S.M., Shin, S.R., and Hassan, S. (2022). Microfluidic fabrication of lipid nanoparticles for the delivery of nucleic acids. Adv. Drug Deliv. Rev. *184*, 114197. https://doi.org/10.1016/j.addr.2022.114197.

53. Goyal, R., Macri, L.K., Kaplan, H.M., and Kohn, J. (2016). Nanoparticles and nanofibers for topical drug delivery. J. Control Release. *240*, 77–92. https://doi.org/10.1016/j.jconrel.2015.10.049.

54. Mai, Y., Ouyang, Y., Yu, M., Qin, Y., Girardi, M., Saltzman, W.M., Cocco, E., Zhao, C., Yu, L., Jia, Y., et al. (2022). Topical formulation based on disease-specific nanoparticles for single-dose cure of psoriasis. J. Control Release. *349*, 354–366. https://doi.org/10.1016/j.jconrel.2022.07.006.

55. Bornert, O., Kocher, T., Gretzmeier, C., Liemberger, B., Hainzl, S., Koller, U., and Nyström, A. (2019). Generation of rabbit polyclonal human and murine collagen VII monospecific antibodies: A useful tool for dystrophic epidermolysis bullosa therapy studies. Matrix Biol. *4*, 100017. https://doi.org/10.1016/j.mbplus.2019.100017.

OMTM, Volume *31*

Supplemental information

CRISPR-Cas9-based non-viral gene editing therapy for topical treatment of recessive dystrophic epidermolysis bullosa

Xianqing Wang, Xi Wang, Yinghao Li, Sigen A, Bei Qiu, Albina Bushmalyova, Zhonglei He, Wenxin Wang, and Irene Lara-Sáez

Supplemental Information

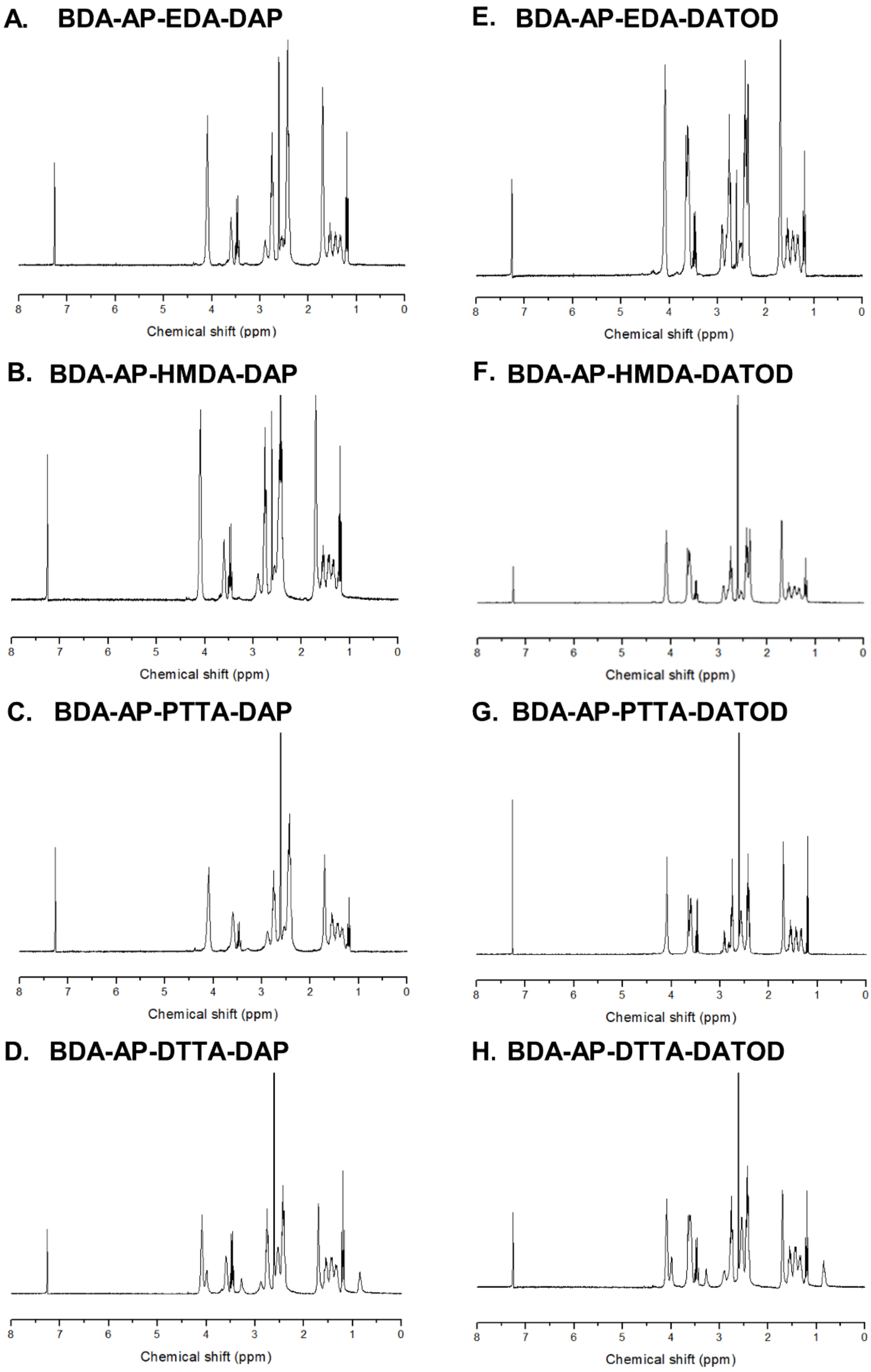

Figure S1 ^{1}H NMR spectrum of HPAEs (400 MHz, CDCl3). (A)BDA-AP-EDA-DAP; (B) BDA-AP-HMDA-DAP; (C)BDA-AP-PTTA-DAP; (D) BDA-AP-DTTA-DAP; (E) BDA-AP-EDA-DATOD; (B) BDA-AP-HMDA-DATOD; (C)BDA-AP-PTTA-DATOD; (D) BDA-AP-DTTA-DATOD.

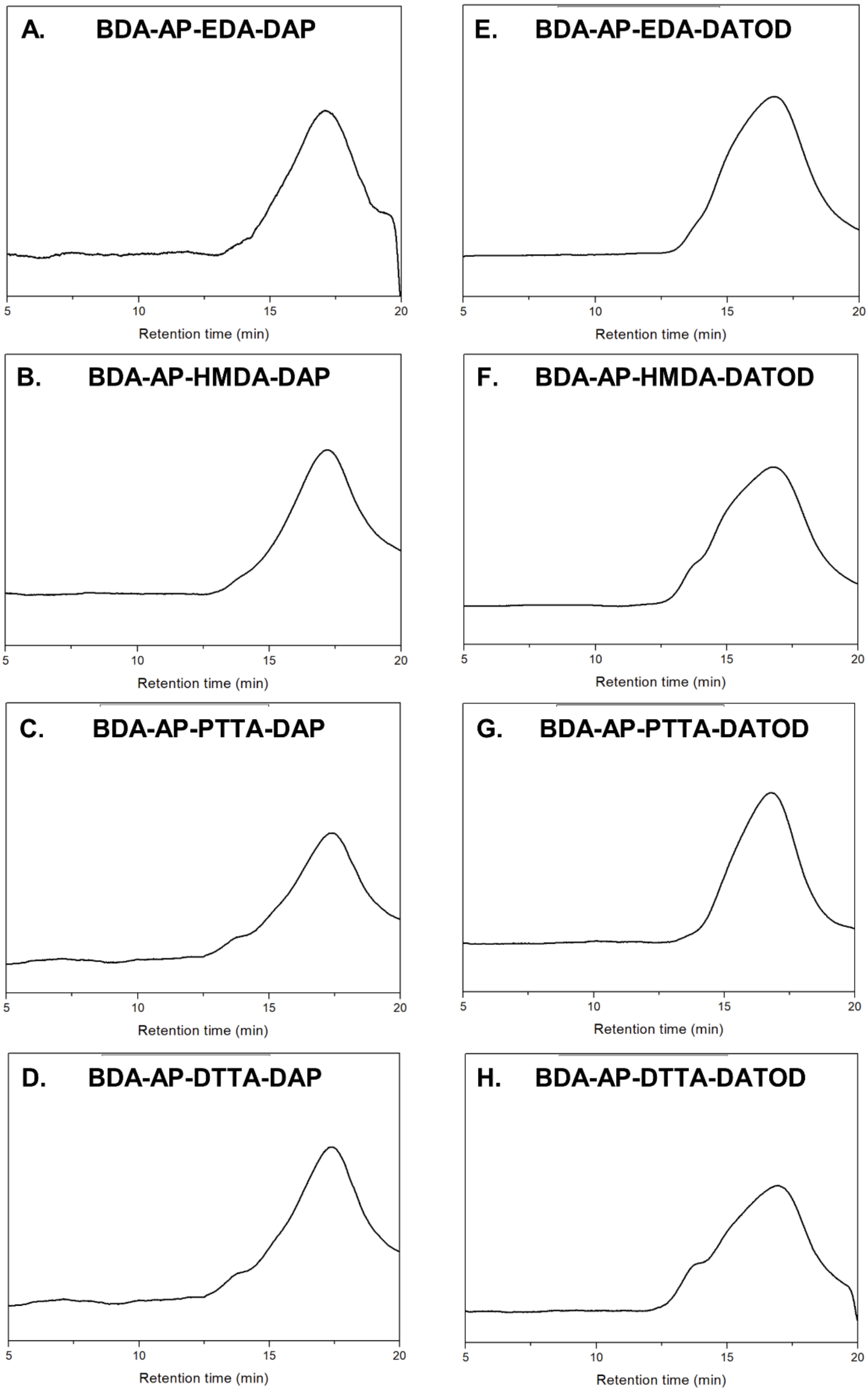

Figure S2 GPC retention time traces of HPAEs. (A)BDA-AP-EDA-DAP; (B) BDA-AP-HMDA-DAP; (C)BDA-AP-PTTA-DAP; (D) BDA-AP-DTTA-DAP; (E) BDA-AP-EDA-DATOD; (B) BDA-AP-HMDA-DATOD; (C)BDA-AP-PTTA-DATOD; (D) BDA-AP-DTTA-DATOD.

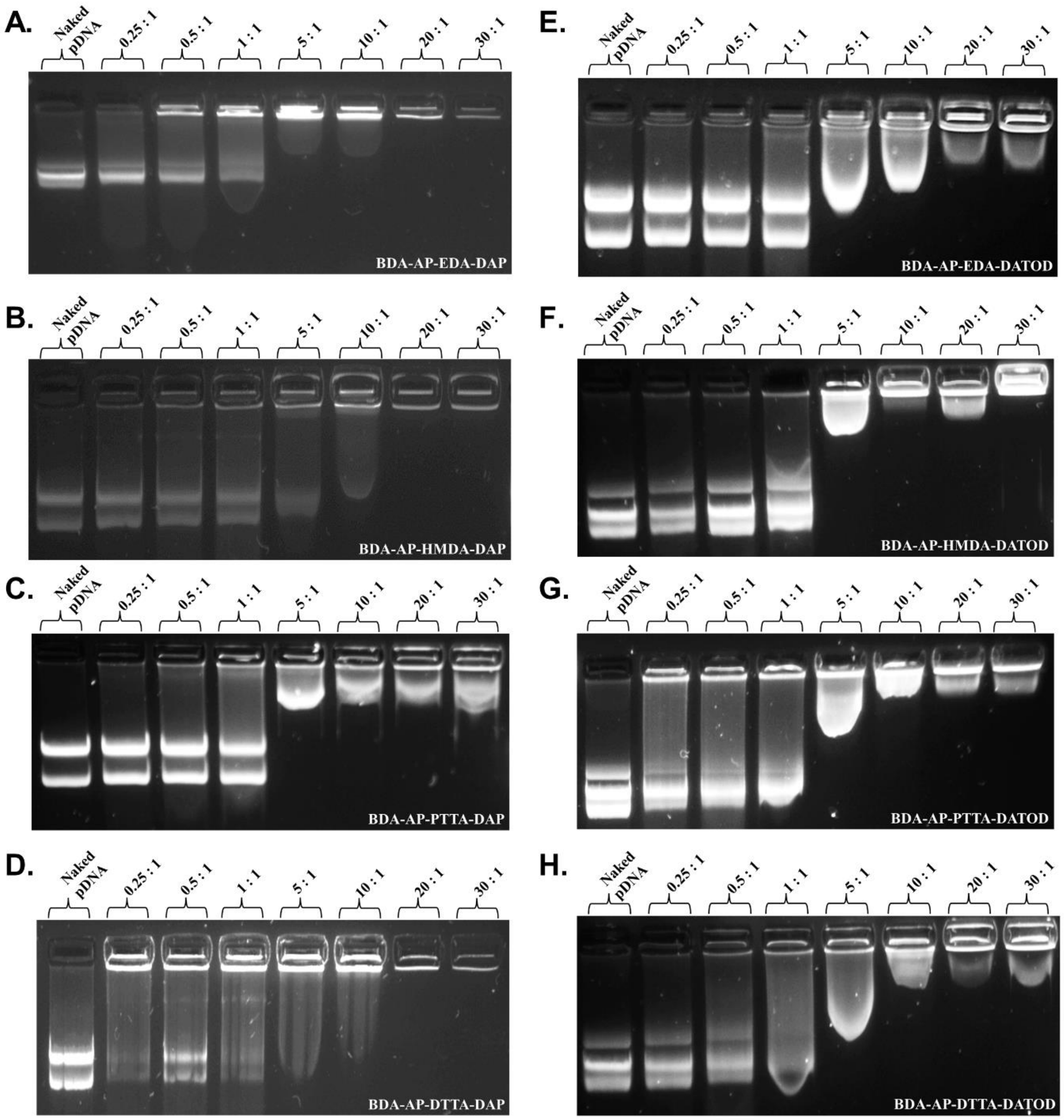

Figure S3 Gel retardation of HPAE/gWiz GFP pDNA. (A)BDA-AP-EDA-DAP; (B) BDA-AP-HMDA-DAP; (C)BDA-AP-PTTA-DAP; (D) BDA-AP-DTTA-DAP; (E) BDA-AP-EDA-DATOD; (B) BDA-AP-HMDA-DATOD; (C)BDA-AP-PTTA-DATOD; (D) BDA-AP-DTTA-DATOD. The DNA binding ability was tested at HPAE to DNA weight ratios from 0.25:1 to 30:1.

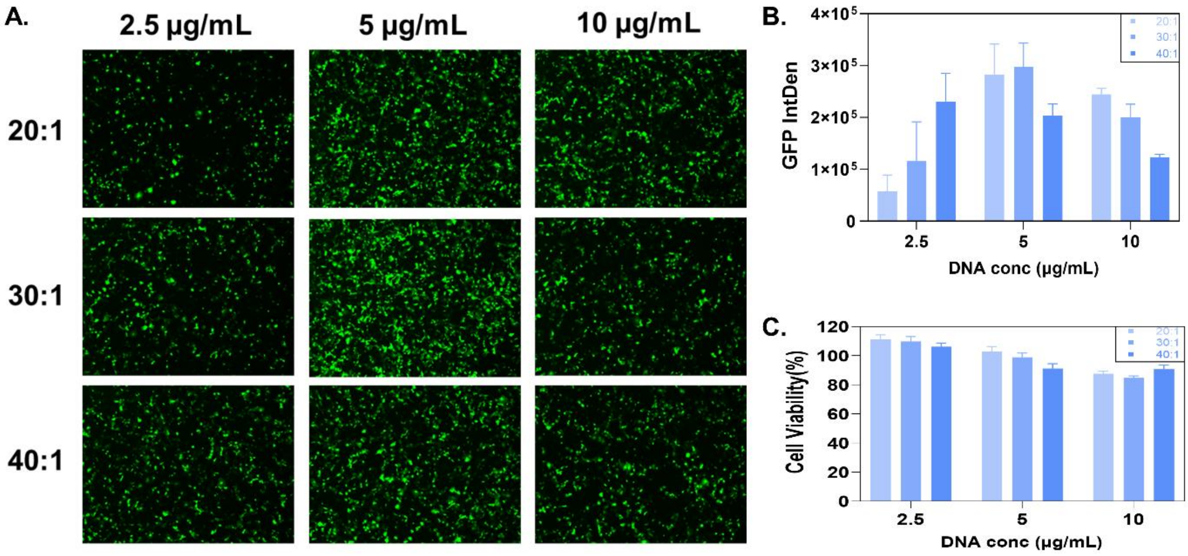

Figure S4 Transfection condition optimization of PTTA-DATOD/GFP pDNA on RDEB keratinocytes. All the data was obtained from 3 individual experiments and presented as Mean ± SD (n=3).

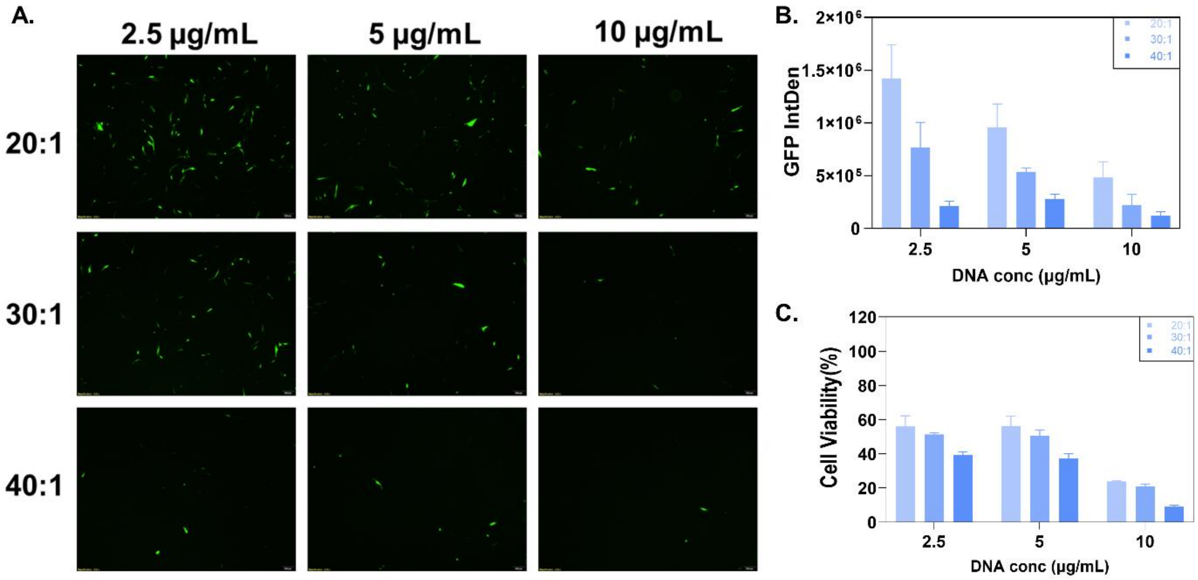

Figure S5 Transfection condition optimization of PTTA-DATOD/GFP pDNA on RDEB Fibroblasts. All the data was obtained from 3 individual experiments and presented as Mean ± SD (n=3).

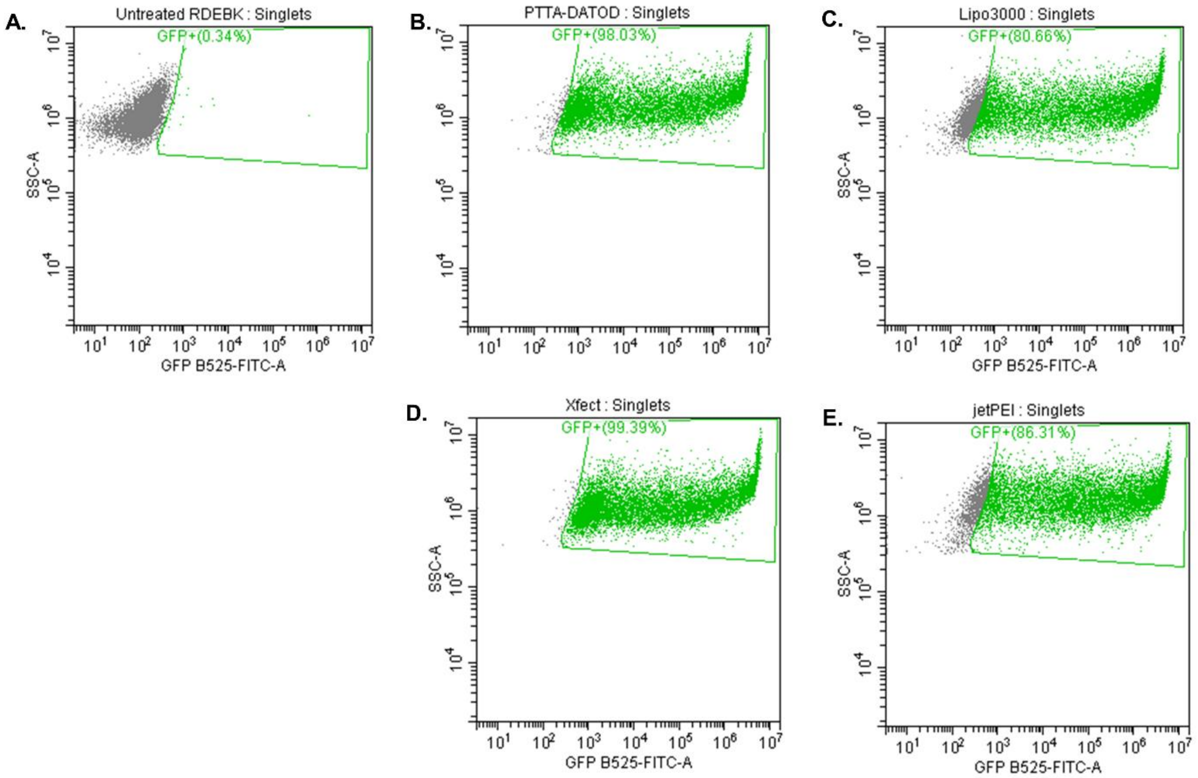

Figure S6 Gating strategy for flowcytometry analysis of RDEBK cells transfected by HPAE/gWiz GFP DNA. (A) Untreated RDEBK cells; (B) RDEBK cells treated with PTTA-DATOD/GFP DNA complexes; (C) RDEBK cells treated with Lipofectamine 3000/GFP DNA complexes; (D) RDEBK cells treated with Xfect/GFP DNA complexes; (E) RDEBK cells treated with jetPEI/GFP DNA complexes.

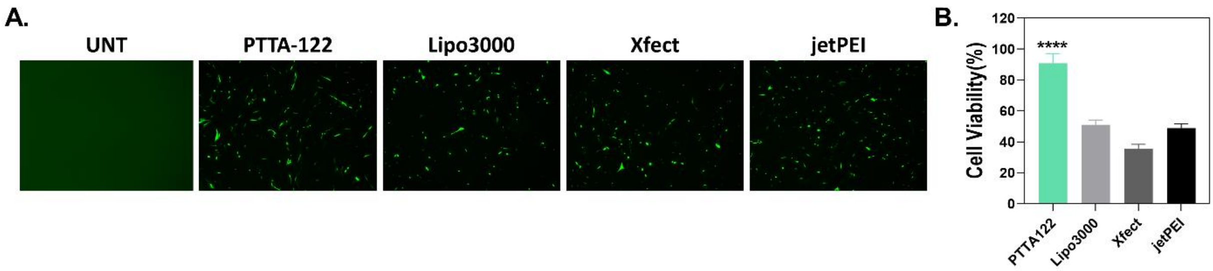

Figure S7 gWiz GFP pDNA transfection in RDEBF cells. (A) Fluorescence images of RDEBF cells transfected by PTTA-DATOD, lipofectamine 3000 (Lipo3000), Xfect and jetPEI (from left to right); (B) Cell viability of treated RDEBF cells. Data was presented as Mean ± SD (n=3).

A.

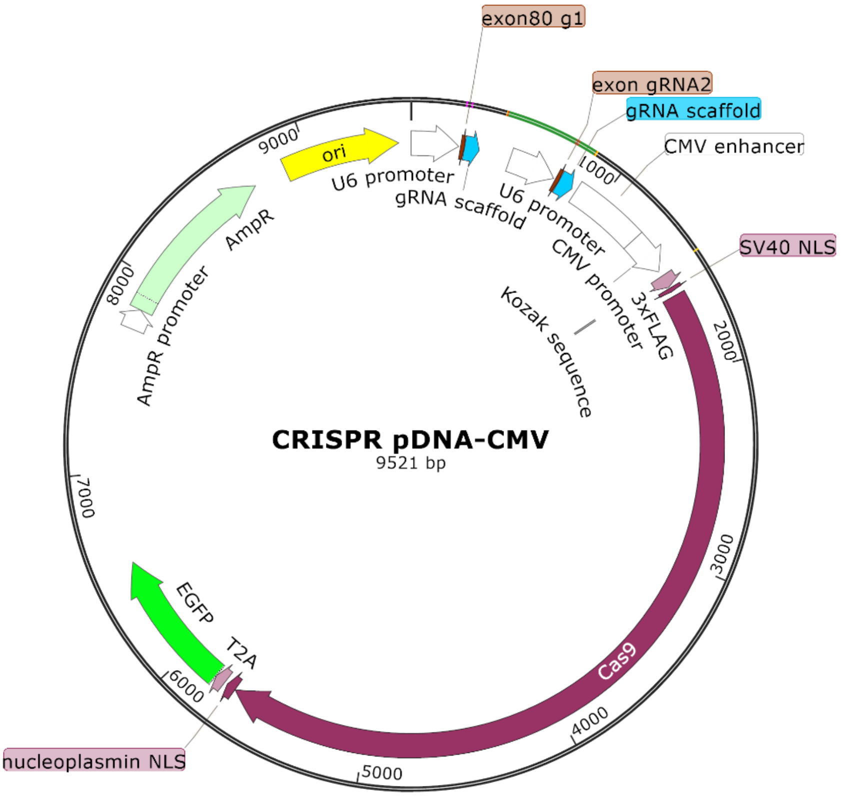

B.

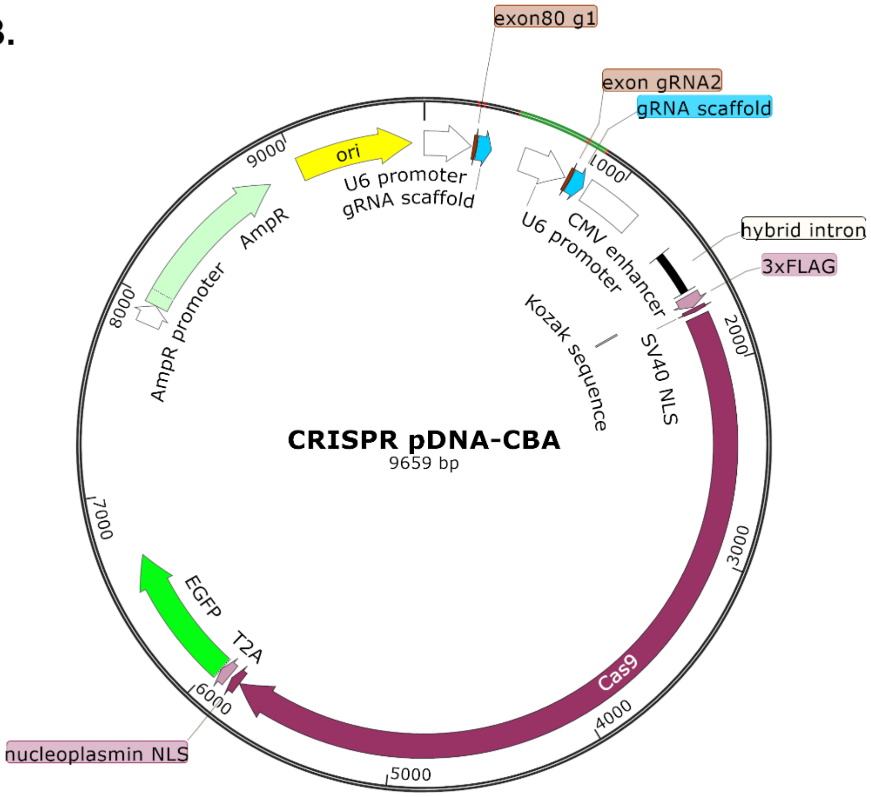

Figure S8 Maps of dual sgRNA guided CRISPR Cas9 plasmids.

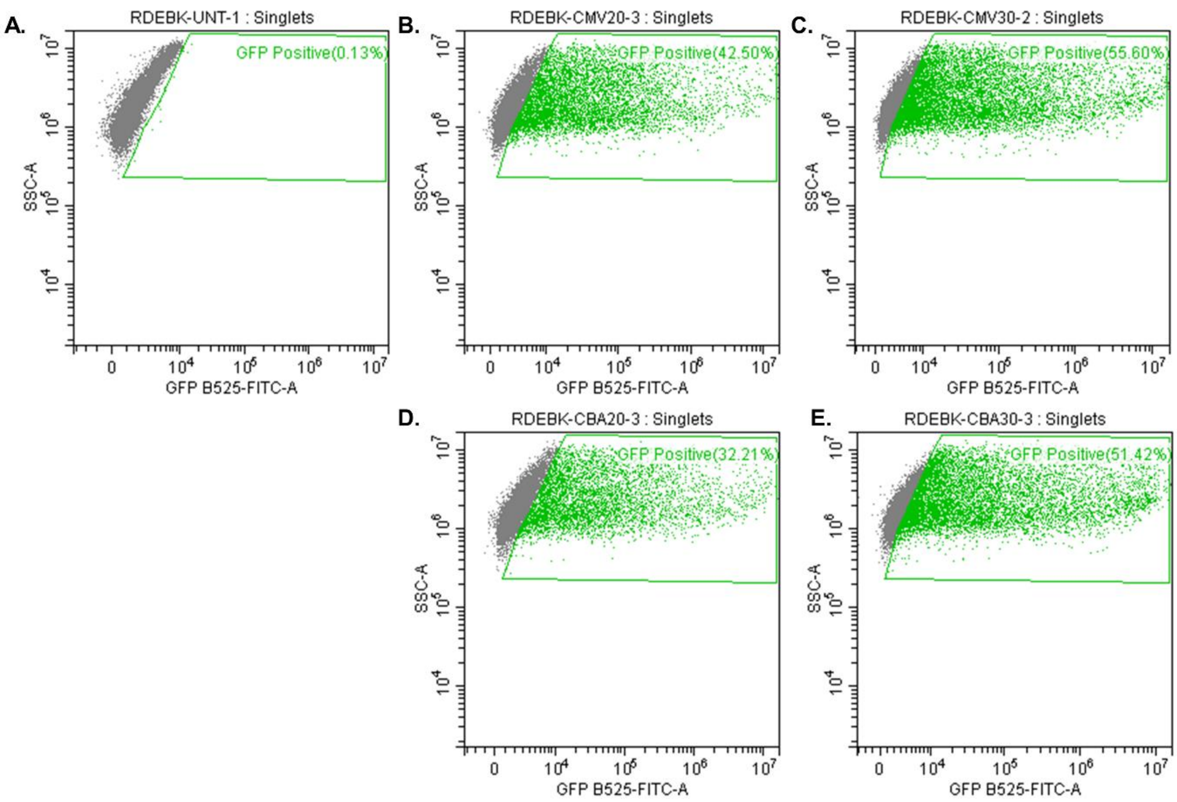

Figure S9 Gating strategy for flowcytometry analysis of RDEBK cells transfected by PTTA-DATOD/CRISPR DNA. (A) Untreated RDEBK cells; (B) RDEBK cells treated with PTTA-DATOD/CRISPR DNA at W/W of 20, the CRISPR DNA has CMV promoter; (C) RDEBK cells treated with PTTA-DATOD/CRISPR DNA at W/W of 30, the CRISPR DNA has CMV promoter; (D) RDEBK cells treated with PTTA-DATOD/CRISPR DNA at W/W of 20, the CRISPR DNA has CBA promoter; (E) RDEBK cells treated with PTTA-DATOD/CRISPR DNA at W/W of 30, the CRISPR DNA has CBA promoter.

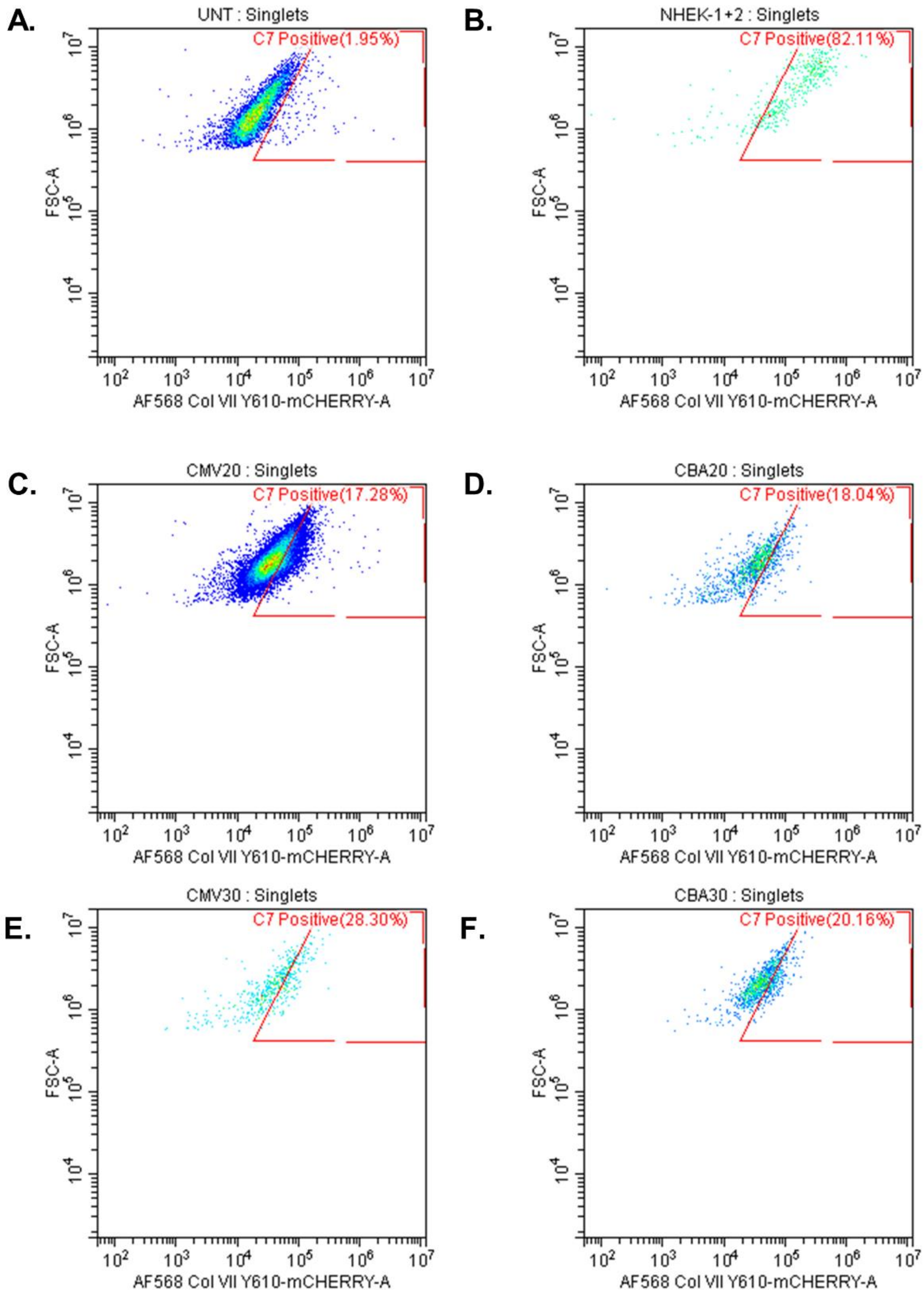

Figure S10 Gating strategy for flow cytometry analysis of type VII collagen expression in cells (A) Untreated RDEBK cells; (B) Human normal epidermal keratinocytes; (C) RDEBK cells treated with PTTA-DATOD/CRISPR DNA at W/W of 20, the CRISPR DNA has CMV promoter; (D) RDEBK cells treated with PTTA-DATOD/CRISPR DNA at W/W of 30, the CRISPR DNA has CMV promoter; (E) RDEBK cells treated with PTTA-DATOD/CRISPR DNA at W/W of 20, the CRISPR DNA has CBA promoter; (F) RDEBK cells treated with PTTA-DATOD/CRISPR DNA at W/W of 30, the CRISPR DNA has CBA promoter.

Table S1 GPC Results

Polymer	Mn	Mw	PDI
BDA-AP-EDA-DAP	2576	8542	3.32
BDA-AP-EDA-DATOD	3481	13408	3.85
BDA-AP-HMDA-DAP	2478	8256	3.33
BDA-AP-HMDA-DATOD	3528	17068	4.84
BDA-AP-PTTA-DAP	2409	10716	4.45
BDA-AP-PTTA-DATOD	3500	10007	2.86
BDA-AP-DTTA-DAP	3297	38016	11.53
BDA-AP-DTTA-DATOD	5172	46832	9.05

Table S2 CRISPR Cas9 sgRNA sequence

sgRNA name	Sequence (5’-3’)
sgRNA 1	CCTGCAGACCCTACATAGAG
sgRNA 2	CAAGACAGGTGAAGGTTCTT

Table S3 TaqMan^{TM} probes for RT-qPCR

Target gene	Probe
Human COL7A1 exon 64	Hs00164310_m1
Human COL7A1 exon 80	Hs01574801_g1
Human GAPDH	Hs02758991_g1
